# Exertional breathlessness related to medical conditions in middle-aged people: the population-based SCAPIS study of more than 25,000 men and women

**DOI:** 10.1186/s12931-024-02766-6

**Published:** 2024-03-16

**Authors:** Magnus Ekström, Josefin Sundh, Anders Andersson, Oskar Angerås, Anders Blomberg, Mats Börjesson, Kenneth Caidahl, Össur Ingi Emilsson, Jan Engvall, Erik Frykholm, Ludger Grote, Kristofer Hedman, Tomas Jernberg, Eva Lindberg, Andrei Malinovschi, André Nyberg, Eric Rullman, Jacob Sandberg, Magnus Sköld, Nikolai Stenfors, Johan Sundström, Hanan Tanash, Suneela Zaigham, Carl-Johan Carlhäll

**Affiliations:** 1 Department of Clinical Sciences Lund, Respiratory Medicine, Allergology and Palliative Medicine, Faculty of Medicine, Lund University, 221 84 Lund, Sweden; 2 Department of Respiratory Medicine, Faculty of Medicine and Health, Örebro University, Örebro, Sweden; 3 COPD Center, Department of Respiratory Medicine and Allergology, Sahlgrenska University Hospital, Gothenburg, Sweden; 4 COPD Center, Department of Internal Medicine and Clinical Nutrition, Institute of Medicine, Sahlgrenska Academy, University of Gothenburg, Gothenburg, Sweden; 5 Department of Molecular and Clinical Medicine, Institute of Medicine, Gothenburg University, Gothenburg, Sweden; 6 Department of Cardiology, Sahlgrenska University Hospital, Gothenburg, Sweden; 7 Department of Public Health and Clinical Medicine, Umeå University, Umeå, Sweden; 8 Department of Molecular and Clinical Medicine, Sahlgrenska Academy, Gothenburg, Sweden; 9 Center for Lifestyle Intervention, Department MGAÖ, Sahlgrenska University Hospital, Gothenburg, Sweden; 10 Department of Clinical Physiology, Karolinska University Hospital, and Karolinska Institutet, Stockholm, Sweden; 11 Department of Clinical Physiology, Sahlgrenska University Hospital, and Sahlgrenska Academy, Gothenburg, Sweden; 12 Department of Medical Sciences, Respiratory, Allergy and Sleep Research, Uppsala University, Uppsala, Sweden; 13 CMIV, Centre of Medical Image Science and Visualization, Linköping University, Linköping, Sweden; 14 Department of Clinical Physiology in Linköping, and Department of Health, Medicine and Caring Sciences, Linköping University, Linköping, Sweden; 15 Department of Community Medicine and Rehabilitation, Physiotherapy, Umeå University, Umeå, Sweden; 16 Center for Sleep and Vigilance Disorders, Institute of Medicine, Sahlgrenska Academy, Gothenburg University, Gothenburg, Sweden; 17 Sleep Disorders Centre, Department of Respiratory Medicine, Sahlgrenska University Hospital, Gothenburg, Sweden; 18 Department of Clinical Sciences, Danderyd University Hospital, Karolinska Institutet, Stockholm, Sweden; 19 Department of Medical Sciences, Clinical Physiology, Uppsala University, Uppsala, Sweden; 20 Department of Laboratory Medicine, Section of Clinical Physiology, Karolinska Institutet, Stockholm, Sweden; 21 Department of Clinical Physiology, Karolinska University Hospital, Stockholm, Sweden; 22 Respiratory Medicine Unit, Department of Medicine Solna and Center for Molecular Medicine, Karolinska Institutet, Stockholm, Sweden; 23 Department of Respiratory Medicine and Allergy, Karolinska University Hospital, Stockholm, Sweden; 24 Department of Medical Sciences, Uppsala University, Uppsala, Sweden; 25 George Institute for Global Health, University of New South Wales, Sydney, Australia; 26 Department of Respiratory Medicine, Skåne University Hospital, Lund University, Malmö, Sweden; 27 Department of Clinical Sciences in Malmö, Lund University, Malmö, Sweden; 28 Center for Medical Image Science and Visualization, Linköping University, Linköping, Sweden

**Keywords:** Dyspnea, Diseases, Obesity, Epidemiology

## Abstract

**Background:**

Breathlessness is common in the population and can be related to a range of medical conditions. We aimed to evaluate the burden of breathlessness related to different medical conditions in a middle-aged population.

**Methods:**

Cross-sectional analysis of the population-based Swedish CArdioPulmonary bioImage Study of adults aged 50–64 years. Breathlessness (modified Medical Research Council [mMRC] ≥ 2) was evaluated in relation to self-reported symptoms, stress, depression; physician-diagnosed conditions; measured body mass index (BMI), spirometry, venous haemoglobin concentration, coronary artery calcification and stenosis [computer tomography (CT) angiography], and pulmonary emphysema (high-resolution CT). For each condition, the prevalence and breathlessness population attributable fraction (PAF) were calculated, overall and by sex, smoking history, and presence/absence of self-reported cardiorespiratory disease.

**Results:**

We included 25,948 people aged 57.5 ± [SD] 4.4; 51% women; 37% former and 12% current smokers; 43% overweight (BMI 25.0–29.9), 21% obese (BMI ≥ 30); 25% with respiratory disease, 14% depression, 9% cardiac disease, and 3% anemia. Breathlessness was present in 3.7%. Medical conditions most strongly related to the breathlessness prevalence were (PAF 95%CI): overweight and obesity (59.6–66.0%), stress (31.6–76.8%), respiratory disease (20.1–37.1%), depression (17.1–26.6%), cardiac disease (6.3–12.7%), anemia (0.8–3.3%), and peripheral arterial disease (0.3–0.8%). Stress was the main factor in women and current smokers.

**Conclusion:**

Breathlessness mainly relates to overweight/obesity and stress and to a lesser extent to comorbidities like respiratory, depressive, and cardiac disorders among middle-aged people in a high-income setting—supporting the importance of lifestyle interventions to reduce the burden of breathlessness in the population.

**Supplementary Information:**

The online version contains supplementary material available at 10.1186/s12931-024-02766-6.

## Background

Activity-related breathlessness [1] affects 10–25% of middle-aged and older people in the general population [2, 3]. The symptom, often defined as a self-rating on the modified Medical Research Council (mMRC) scale of ≥ 2 (‘I walk slower than people of the same age on the level because of breathlessness or have to stop for breath when walking at my own pace on the level’, or worse) [4], associates strongly with impaired physical function and activity [5], fatigue [6], worse quality of life [7], and premature death [8].

Activity-related breathlessness (henceforth ‘breathlessness’) arises in response to an increased ventilatory drive (need to breathe), and/or decreased ventilatory capacity (ability to breathe), and central brain processing (involving personality traits, emotional and circumstantial factors) [1, 9]. Medical conditions that affect any of the ventilatory drive, capacity, or the central brain processing of the symptom can all cause or aggravate breathlessness [1], such as cardiorespiratory disease, overweight and obesity, depression, stress, and anemia [3, 10–14]. While the pathophysiological links between these medical conditions and increased exertional breathlessness are well established in laboratory studies [1, 15, 16], data on the epidemiology of breathlessness in the population are surprisingly scarce [10].

Knowledge is limited on the contribution of medical conditions to the burden of breathlessness in the population. To date, evidence pertains to an interview study of 268 people with breathlessness, of whom about 70% attributed the symptom to underlying respiratory disease [17]. In a small Swedish single centre study of middle-aged people (n = 108) with mostly mild to moderate breathlessness, the most prevalent medical conditions were respiratory disease (57%), anxiety or depression (52%), obesity (43%), and heart disease (35%), with two or more conditions present in 66% [3], and similar findings have been reported from Canadian [18] and Australian [19] surveys. In a recent analysis, obesity contributed to about 22% of breathlessness cases in a sample of Australian adults, expressed as a population attributable fraction (PAF) of 21–24% [11].

However, no study has evaluated the breathlessness burden and PAFs related to different medical conditions in a large population study, with data including physiological measurements. Improved data on the epidemiology of breathlessness and contributing medical conditions, including differences by sex, smoking history, and in people without self-reported (known) cardiorespiratory disease, are important to inform the clinical evaluation and management of breathlessness and public health interventions.

We aimed to evaluate the burden of breathlessness in the middle-aged general population related to different medical conditions. Secondary aims were to evaluate breathlessness in clinically relevant subgroups by sex, smoking history, and presence/absence of self-reported cardiorespiratory disease.

## Methods

### Study design and population

This was a population-based, multicentre, cross-sectional analysis of the Swedish CArdioPulmonary bioImage Study (SCAPIS; www.scapis.org) of men and women aged 50 to 64 years [20]. The study design and assessments have been detailed elsewhere [20]. SCAPIS collected data between 2013–2018 at six study centres (Gothenburg, Linköping, Malmö/Lund, Stockholm, Umeå, and Uppsala). To be eligible, participants had to be able to understand instructions and complete questionnaires in Swedish. The SCAPIS sample has been found to be representative of the age-matched Swedish general population [21].

Exclusion criteria in the present study were missing data on breathlessness (mMRC) or on any of the medical conditions or confounders in the analysis (specified below); or inability to walk for reasons other than breathlessness (as the outcome relates to breathlessness on walking).

This analysis extends a previous exploration of underlying conditions in people with breathlessness in the SCAPIS pilot study (n = 1097) [3], which is not included in the present database. This study is reported in accordance with the STrengthening the Reporting of OBservational studies in Epidemiology statement [22].

### Assessments

Definitions of all conditions in the analyses are found in Additional file 1: Table S1.

#### Self-reported data

Breathlessness was defined as a self-rated mMRC score of ≥ 2 (‘I walk slower than people of the same age on the level because of breathlessness or have to stop for breath when walking at my own pace on the level’, or worse) [4]. This cut-off reflects breathlessness affecting everyday life [23], is the most specific threshold to identify people with abnormally increased breathlessness on standardized exercise testing [24], and is endorsed by clinical guidelines [25].

Other self-reported data included: demographics; smoking history (never, former, current daily smoking); pack-years of smoking; highest completed education (university, secondary, primary school, or none); type of residence (own house, own apartment, rented apartment, or other); and self-reported physician-diagnosed conditions, stress, depression, and physical exercise level (Additional file 1: Table S1). Stress was assessed using the question: “By stress we mean feeling tense, irritable, anxious or having sleeping difficulties as a result of conditions at work or at home. Did you experience this?”, and was self-rated on a 5-point ordinal scale in accordance with Rosengren et al*.* [26, 27] as: 0 “never”, 1 “any stress period”, 2 “some stress periods during the last five years”, 3 “constant stress during the last year”, and 4 “constant stress during the last five years”. In the analyses, the presence of stress was categorized as a score of ≥ 2. Depression was defined, according to Rosengren et al*.* [27], as an affirmative answer to ‘During the past twelve months, was there ever a time when you felt sad, blue, or depressed for two weeks or more in a row?’, together with affirming at least five of the seven related questions of having: (1) ‘Lost interest in most things like hobbies, work or activities that usually give you pleasure?’; (2) ‘Felt tired or low on energy?’; (3) ‘Gained or lost weight?’; (4) ‘Trouble falling asleep?’; (5) ‘Concentration problems?’; (6) ‘Thoughts about death?’; and (7) ‘Bad self-esteem/feeling worthless?’.

#### Measured data

Assessments were performed at a baseline visit and included body mass index (BMI), with overweight defined as a BMI 25–29.9, and obesity as a BMI ≥ 30 kg/m^2^; post-bronchodilator spirometry forced expiratory volume in one second (FEV_1_) and forced vital capacity (FVC), evaluated using Global Lung Function Initiative references [28, 29]. Chronic airflow limitation (CAL) was defined as a post-bronchodilator FEV_1_/FVC < 0.7 [25]. Restrictive spirometry pattern was defined as having a FVC < lower limit of normal (LLN) and FEV_1_/FVC > LLN.

As detailed in Additional file 1: Table S1, pulmonary emphysema was assessed using high-resolution computed tomography (HRCT); coronary artery calcifications (CAC) using non-contrast computed tomography CT; presence of coronary artery stenosis was assessed using coronary computed tomography angiography (CCTA); venous N-terminal pro b-type natriuretic peptide (NT-proBNP; ng/ml) and anemia [haemoglobin (Hb) < 120 g/L (12 g/dL) in women and < 130 g/L (13 g/dL) in men] were assessed.

#### Diagnosis groups

Respiratory diseases were defined as: self-reported physician-diagnosed asthma, chronic bronchitis, chronic rhinosinusitis, other respiratory disease; spirometric CAL, restrictive spirometry pattern, or emphysema on HRCT.

Cardiac diseases were defined as: self-reported physician-diagnosed atrial fibrillation/flutter, ischemic heart disease [defined as previous myocardial infarction, coronary artery bypass graft or percutaneous coronary intervention (CABG/PCI)], cardiac valvular disease, heart failure, ischemic heart disease; and coronary artery stenosis on CT angiography.

For the subgroup analyses, the presence of self-reported cardiorespiratory disease was defined as any self-report of physician-diagnosed asthma, chronic obstructive pulmonary disease (COPD), chronic bronchitis, chronic rhinosinusitis, other respiratory disease, atrial fibrillation/flutter, cardiac valvular disease, heart failure, or ischemic heart disease.

### Statistical analyses

Medical conditions to be evaluated were selected based on the authors’ subject matter knowledge and the breathlessness literature, comprising conditions related to activity-related breathlessness in mechanistic [1, 15, 16] and population studies [2, 10, 11, 13, 18, 30, 31].

The prevalence of each condition was tabulated between people with/without breathlessness and in subgroups. Number of concurrent conditions in each participant was calculated and overlap between the most common conditions was presented using Venn diagrams.

Association for each condition with breathlessness was analysed as odd ratios (ORs) using logistic regression, with 95% confidence intervals (CIs) accounting for clustering by study centre. All regression models were performed unadjusted and adjusted for potential confounders (based on a directed acyclical graph; www.dagitty.net, Additional file 1: Figure S1): age, sex, smoking history, pack-years of smoking, highest completed education, and BMI (when appropriate). No data were imputed. As the exercise level could be affected by both different conditions and breathlessness (being a potential collider variable), associations with self-reported exercise were evaluated in the fully adjusted model separately.

PAFs, fractions of breathlessness cases in the population attributable to each medical condition, were calculated based on the prevalence of each condition and its adjusted association (aOR) with breathlessness in accordance with Miettinen [32]. A sensitivity analysis using the unadjusted ORs yielded similar estimates. A PAF of 25% could be interpreted as that ¼ of the population risk of breathlessness is related to the factor and would be prevented if the medical condition was removed, assuming that all confounding factors had been accounted for [33]. However, as residual confounding cannot be excluded, PAFs should be interpreted as a measure of a factor’s relation to the burden of breathlessness on the population level [32]. As there can be many potentially contributing conditions, PAFs can sum to more than 100% [33].

All analyses were performed for all participants, and for subgroups by: (1) sex; (2) smoking history (never-, former, or current smokers); and (3) presence of any self-reported cardiorespiratory disease. All estimates were reported with 95% confidence intervals (CIs). Statistical analyses were conducted using the software packages Stata, version 17.0 (StataCorp LP; College Station, TX).

## Results

### Participants and breathlessness

After excluding people with missing data (n = 4018; for details see Additional file 1: Table S2) or inability to walk for reasons other than breathlessness (n = 188), a total of 25,948 people were included in the analyses. Included people were 51% women and had a mean age of 57.5 years (SD 4.4), mean BMI 26.9 kg/m^2^ (SD 4.4), and 49% were current or former smokers (Table 1; Additional file 1: Table S2).

**Table 1:** Characteristics of participants by the presence of breathlessness

	Without breathlessness (mMRC 0–1)	With breathlessness (mMRC ≥ 2)
N	24,996 (96.3%)	952 (3.7%)
Age (years)	57.4 (4.3)	58.3 (4.4)
Female sex	12,619 (50.5%)	646 (67.9%)
mMRC breathlessness rating		
0	23,741 (95.0%)	0
1	1255 (5.0%)	0
2	0	773 (81.2%)
3	0	88 (9.2%)
4	0	91 (9.6%)
Smoking history		
Never	12,987 (52.0%)	361 (37.9%)
Former	9092 (36.4%)	414 (43.5%)
Current	2917 (11.7%)	177 (18.6%)
Pack-years of smoking	7.2 (11.4)	14.2 (17.2)
Body mass index (kg/m^2^)		
< 25.0	9255 (37.0%)	128 (13.4%)
25–29.9	10,957 (43.8%)	293 (30.8%)
≥ 30	4784 (19.1%)	531 (55.8%)
FEV_1_ (l)	3.3 (0.8)	2.7 (0.7)
FEV_1_ (%pred)	102.9 (13.4)	92.5 (17.0)
FVC (l)	4.3 (1.0)	3.6 (0.9)
FVC (%pred)	103.3 (12.7)	96.1 (13.9)
FEV_1_/FVC	0.8 (0.1)	0.8 (0.1)
FEV_1_/FVC (%pred)	99.3 (7.6)	95.8 (11.6)
Highest completed education		
University	11,692 (46.8%)	276 (29.0%)
Secondary	11,199 (44.8%)	494 (51.9%)
Primary or none	2051 (8.2%)	180 (18.9%)
Missing	54 (0.2%)	2 (0.2%)
Residence		
Own house	12,105 (48.4%)	329 (34.6%)
Own apartment	7447 (29.8%)	277 (29.1%)
Rented apartment	5252 (21.0%)	334 (35.1%)
Other	176 (0.7%)	12 (1.3%)
Missing	16 (0.1%)	0 (0.0%)
Self-reported exercise level		
> 3 times/week	3215 (12.9%)	47 (4.9%)
2–3 times/week	4466 (17.9%)	51 (5.4%)
1–2 times/week	5199 (20.8%)	128 (13.4%)
Not regularly	5374 (21.5%)	224 (23.5%)
Never	6613 (26.5%)	491 (51.6%)
Missing	129 (0.5%)	11 (1.2%)

Data are presented as mean (standard deviation) or frequency (%)

*BMI* body mass index, *FEV*_*1*_ forced expiratory volume in one second, *FVC* forced vital capacity, *mMRC* modified Medical Research Council, *pred* predicted value, *SD* standard deviation

Compared to people who were excluded from the analyses (Additional file 1: Table S2), the included people tended to have smoked less, have higher socioeconomic status in terms of education level and residence status, and to have a somewhat lower prevalence of respiratory disease and depression.

### Prevalence of medical conditions in relation to breathlessness

Breathlessness (mMRC ≥ 2) was present in 952 (3.7%) of people. The distribution of breathlessness ratings are shown in Table 1. Compared with people without breathlessness, people with breathlessness were more women and had higher rates of previous or current smoking, obesity, lung function impairment, and lower socioeconomic status (education level and residence status) (Table 1).

Prevalence of the medical conditions in the population is shown in Table 2. People with breathlessness had a higher prevalence of obesity (56% vs. 19%), respiratory disease (49% vs. 24%), stress (with 41% vs. 20% reporting constant stress for one year or longer), depression (37% vs. 13%), cardiac disease (19% vs. 9%), and anemia (5% vs. 3%), compared to people without breathlessness (Table 2); p < 0.001 for all comparisons. In the study population, the most common respiratory conditions were asthma, CAL, emphysema, and chronic bronchitis. The most common cardiac conditions were coronary artery stenosis, ischemic heart disease, and atrial fibrillation/flutter (Table 2). NT-proBNP (in the 25,286 people with data) was higher in people with breathlessness (median 61.4 ng/ml; IQR 34.0–109.1) than in people without breathlessness (median 47.6 ng/ml; IQR 28.8–79.1; p < 0.001 using ranksum test). The prevalence of a NT-proBNP ≥ 300 ng/ml was low overall but higher in people with breathlessness (6.0% vs. 1.3%; p < 0.001). In people without any cardiac condition, the corresponding prevalence of a NT-proBNP ≥ 300 ng/ml was 2.7% vs. 0.8%.

**Table 2:** Medical conditions in relation to breathlessness in the middle-aged general population

	Without breathlessness (mMRC 0–1)	With breathlessness (mMRC ≥ 2)	Association with breathlessness Odds ratio (95% CI)	
			Crude	Adjusted for confounders*
	N = 24,996	N = 952	N = 25,948**	N = 25,948**
Respiratory disease	5994 (24.0%)	470 (49.4%)	3.1 (2.5–3.8)	2.7 (2.1–3.6)
Asthma	1836 (7.3%)	189 (19.9%)	3.1 (2.6–3.7)	2.9 (2.5–3.4)
Chronic airflow limitation	2507 (10.0%)	199 (20.9%)	2.4 (2.1–2.7)	2.3 (1.9–2.7)
Emphysema (HRCT)	1314 (5.3%)	113 (11.9%)	2.4 (1.8–3.2)	1.9 (1.3–2.9)
Chronic bronchitis	604 (2.4%)	103 (10.8%)	4.9 (4.1–5.9)	4.2 (3.3–5.2)
Chronic rhinosinuitis	513 (2.1%)	51 (5.4%)	2.7 (1.7–4.4)	2.3 (1.2—4.4)
Restrictive spirometry pattern	415 (1.7%)	51 (5.4%)	3.4 (2.6–4.3)	2.7 (2.0–3.5)
Other respiratory disease	277 (1.1%)	43 (4.5%)	4.2 (3.0–5.9)	4.2 (3.0–5.9)
Body mass index (kg/m^2^)				
< 25.0	9,255 (37.0%)	128 (13.4%)	1 (Ref)	1 (Ref)
25–29.9	10,957 (43.8%)	293 (30.8%)	1.9 (1.7–2.2)	2.1 (1.8–2.4)
≥ 30	4,784 (19.1%)	531 (55.8%)	8.0 (7.3–8.9)	7.6 (6.7–8.6)
Anemia	837 (3.3%)	49 (5.1%)	1.6 (1.1–2.1)	1.8 (1.3–2.4)
Peripheral arterial insufficiency	65 (0.3%)	8 (0.8%)	3.3 (2.1–5.0)	3.0 (2.1–4.1)
Stress level				
Never stress	1263 (5.1%)	22 (2.3%)	1 (Ref)	1 (Ref)
Any stress period	9006 (36.0%)	255 (26.8%)	1.6 (0.9–3.1)	1.9 (1.0–3.8)
Some stress periods (last 5 yrs)	9799 (39.2%)	283 (29.7%)	1.7 (1.0–2.8)	2.2 (1.2–4.0)
Constant stress (last 1 year)	2479 (9.9%)	146 (15.3%)	3.4 (2.1–5.4)	4.1 (2.4–7.2)
Constant stress (last 5 yrs)	2449 (9.8%)	246 (25.8%)	5.8 (3.4–10.0)	6.7 (3.8–11.8)
Depression	3350 (13.4%)	350 (36.8%)	3.8 (3.1–4.6)	2.9 (2.3–3.5)
Cardiac disease	2204 (8.8%)	178 (18.7%)	2.4 (2.9–3.9)	2.3 (1.7–3.2)
Coronary artery stenosis	1301 (5.6%)	76 (9.3%)	1.7 (1.57–2.1)	1.6 (1.4–1.9)
Ischemic heart disease	447 (1.8%)	53 (5.6%)	3.2 (2.6–4.1)	2.3 (1.7–3.2)
CABG/PCI	242 (1.0%)	24 (2.5%)	2.6 (1.8–3.9)	2.0 (1.2–3.4)
Myocardial infarction	351 (1.4%)	45 (4.7%)	3.5 (2.7–4.6)	2.4 (1.7–3.4)
Atrial fibrillation/flutter	417 (1.7%)	41 (4.3%)	2.7 (1.9–3.7)	2.6 (1.8–3.8)
Cardiac valvular disease	184 (0.7%)	23 (2.4%)	3.3 (1.5–7.5)	3.9 (1.7–9.2)
Heart failure	93 (0.4%)	26 (2.7%)	7.5 (4.2–13.4)	6.1 (3.2–11.8)
CAC total score	0.0 (0.0–19.0)	0.0 (0.0–42.0)	–	–
CAC category				
0	14,638 (58.6%)	489 (51.4%)	1 (Ref)	1 (Ref)
1–100	6994 (28.0%)	267 (28.0%)	1.1 (1.0–1.3)	1.0 (0.9–1.1)
> 100	2851 (11.4%)	152 (16.0%)	1.6 (1.3–2.0)	1.3 (1.1–1.6)
Missing	513 (2.1%)	44 (4.6%)	–	–

Data presented as frequency (%) and associations as odds ratio (95% confidence interval) analyzed using logistic regression. CAL was defined as a ratio of the forced expired volume in 1 s to the forced vital capacity (FEV_1_/FVC) < 0.7 post bronchodilator. Restrictive spirometry pattern was defined as having a FVC < the lower limit of normal (LLN) and FEV_1_/FVC ≥ LLN

*CABG* coronary artery bypass graft surgery, *CAC* coronary artery calcification, *CI* confidence interval, *HRCT* high resolution computer tomography, *mMRC* modified Medical Research Council breathlessness score, *PCI* percutaneous coronary intervention

^*^Adjusted for age, sex, smoking history, pack-years of smoking, highest completed education, and body mass index (when appropriate)

^**^The analysis of coronary artery stenosis included 25,391 people due to missing data for the variable (n = 557)

**Fig. 1:**
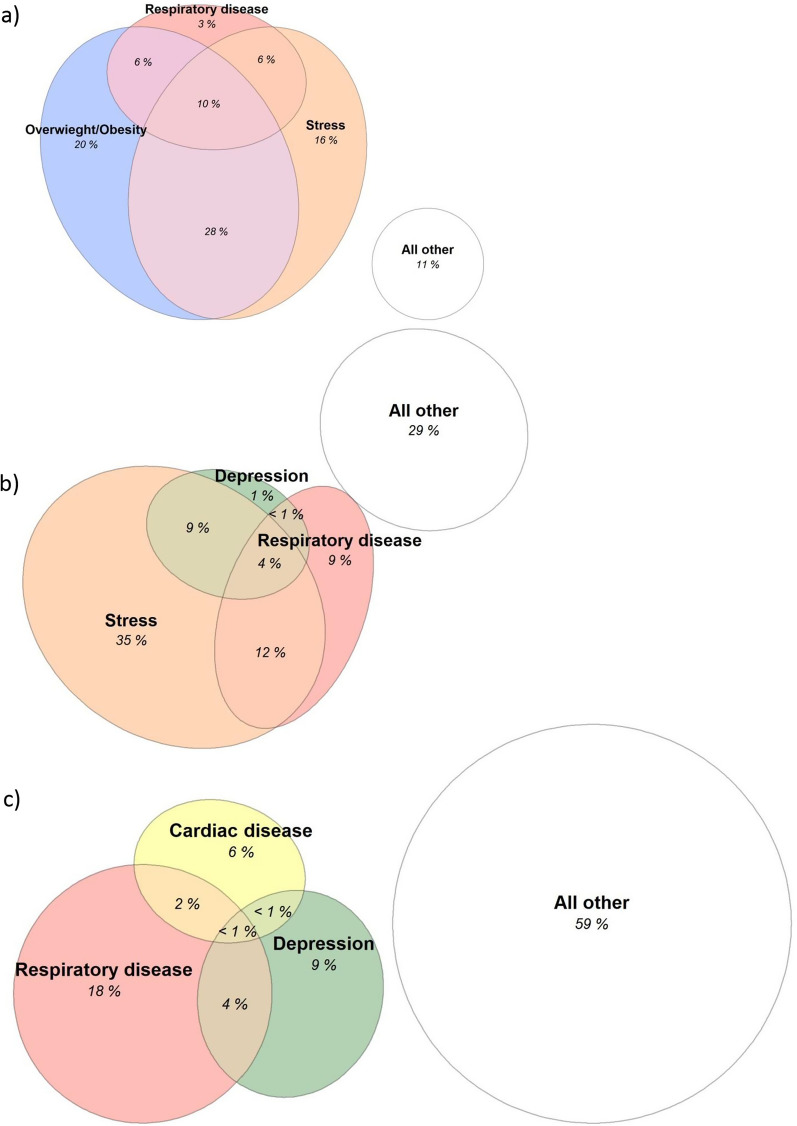
Overlap between the most common medical conditions related to breathlessness in the middle-aged population: **a** respiratory disease, stress, and obesity; **b** respiratory disease, stress, and depression; and **c** respiratory disease, depression, and cardiac disease

Underlying conditions often coexisted; the median number of conditions per participant (of the maximum 19 conditions listed in Table 2) was 2 (IQR 2–3; range 0–12). The degree of overlap between the most common conditions is shown in Fig. 1.

All the evaluated medical conditions were associated with increased breathlessness prevalence in the population, also after adjusting for confounders (Table 2). The strongest associations with increased breathlessness (in order of magnitude) were seen for obesity (aOR 7.6), stress (aOR 6.7), heart failure (aOR 6.1), and chronic bronchitis (aOR 4.2). Overweight and obesity remained strongly associated with breathlessness when adjusting for confounders as well as all medical conditions in Table 2: overweight aOR 2.3 ([95% CI], 2.0–2.8), and obesity aOR 8.2 (6.7–10.1). In the same model, the associations for the other main factors were attenuated but remained: stress (aOR 3.9; 2.1–7.3), heart failure (aOR 2.6; 1.4–4.7), and chronic bronchitis (aOR 2.7; 2.0–3.8).

Lower self-reported exercise level associated with higher breathlessness prevalence; exercising 1–2 times/week (aOR 1.4; [95% CI] 1.0–1.8), not regularly (aOR 1.8; 1.5–2.1), and never (aOR 2.8; 2.2–3.6), compared with people who exercised > 3 times/week. When adjusting the models for self-reported exercise level, in addition to the confounders, findings were similar (data not shown).

### Population attributable fractions

The fraction of breathlessness cases in the study population related to each medical condition is shown in Table 3. The medical conditions with the highest PAFs for breathlessness were (in order of magnitude): overweight and obesity ([95% CI] 59.6–66.0%), stress (31.6–76.8%), respiratory disease (20.1–37.1%), depression (17.1–26.6%), cardiac disease (6.3–12.7%), and anemia (0.8–3.3%).

**Table 3:** Population attributable fraction (PAF) of breathlessness related to underlying medical conditions by order of magnitude

Condition	PAF	95% confidence interval
Overweight and obesity	62.9%	59.6–66.0%
Stress	60.2%	31.6–76.8%
Respiratory disease	29.1%	20.1–37.1%
Asthma	11.9%	9.8–14.0%
Chronic airflow limitation	10.5%	7.6–13.4%
Chronic bronchitis	7.4%	5.7–9.1%
Emphysema (on HRCT)	5.1%	1.3–8.6%
Chronic rhinosinuitis	2.7%	0–5.5%
Restrictive spirometry pattern	3.0%	1.9–4.2%
Other respiratory disease	3.1%	2.1–4.1%
Depression	22.0%	17.1–26.6%
Cardiac disease	9.5%	6.3–12.7%
Coronary artery stenosis	3.2%	1.8–4.6%
Ischemic heart disease	2.7%	1.5–4.0%
Myocardial infarction	2.3%	1.1–3.5%
CABG/PCI	1.1%	0.1–2.1%
Atrial fibrillation/flutter	2.4%	1.0–3.8%
Heart failure	2.1%	0.9–3.2%
Valvular disease	1.7%	0.1–3.3%
CAC	4.3%	0.4–7.9%
Anemia	2.0%	0.8–3.3%
Peripheral arterial disease	0.5%	0.3–0.8%

The PAF is interpreted as the proportion of cases of breathlessness that would disappear if the condition was theoretically removed from the population all other factors being similar. As conditions coexist, PAFs may not sum to 100%. CAC was defined as any coronary artery calcification (CAC total score > 0 vs. 0)

*CABG* coronary artery bypass graft surgery, *CAC* coronary artery calcifications, *HRCT* high resolution computer tomography, *PCI* percutaneous coronary intervention

^*^Adjusted for age, sex, smoking history, pack-years, highest education and BMI (except when BMI is analyzed as factor)

### Subgroups

**Fig. 2:**
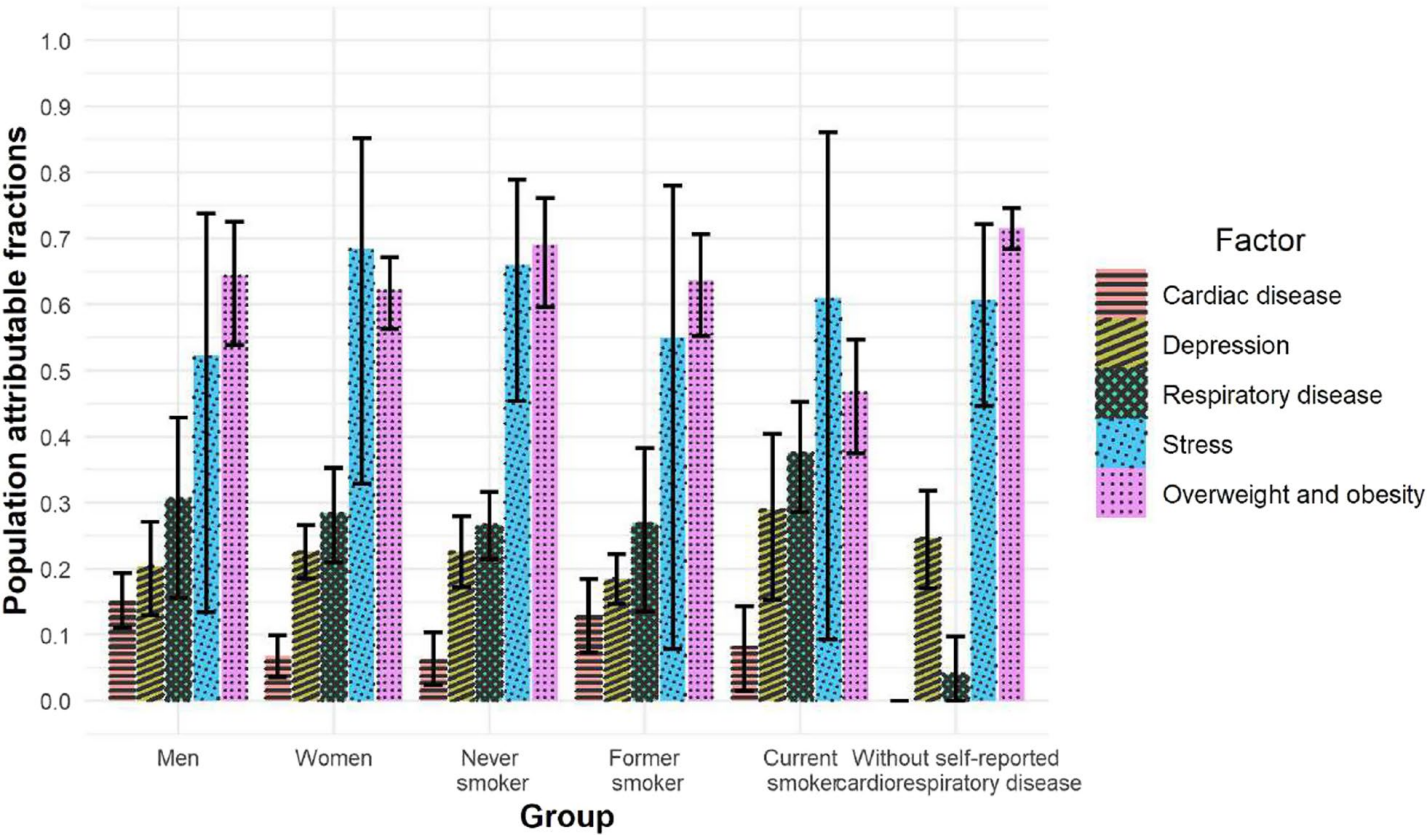
Population attributable fraction (PAF) of breathlessness related to medical conditions among middle-aged people. The PAF reflects the burden of breathlessness that is related to each medical condition, and is interpreted as the proportion of cases of breathlessness [defined as a modified Medical Research Council (mMRC) rating ≥ 2] that would be reduced if the medical condition were to be entirely removed from the population (all other risk factors being similar). PAFs (with 95% confidence intervals) are reported by sex, smoking history, and for people without self-reported cardiorespiratory disease. The group with self-reported cardiorespiratory disease is not included as estimates were less informative (due to the selection criterion)

All analyses were performed separately by sex (Additional file 1: Tables S3–S10), smoking history (Additional file 1: Tables S11–S21), and by presence of self-reported cardiorespiratory disease (Additional file 1: Tables S22–S29). The PAFs of breathlessness related to each medical condition for each subgroup are shown in Fig. 2. Main findings for the subgroups were:

#### By sex

Compared with men, women tended to have more breathlessness (5% vs. 2%), lower BMI, more stress, depression (19.2% vs. 9.1%), and anemia (4.6% vs*.* 2.2%), but less cardiac disease (Additional file 1: Table S3). Associations with breathlessness were similar between the sexes. In men, PAFs were similar as to in the main analysis (whole population), whereas in women the condition contributing to most cases of breathlessness was stress (32.8–85.2%; Additional file 1: Table S10).

#### By smoking history

Breathlessness was present in 5.7% of current smokers, 4.3% of former, and 2.7% of never-smokers. Respiratory disease was more common in current smokers (40%) than in former (27%) and never-smokers (20%), mainly driven by a higher prevalence of CAL in current and former smokers (Additional file 1: Table S12). Smokers also had a higher prevalence of coronary artery stenosis (8.1% in current, 6.5% in former, compared with 4.5% in never-smokers), whereas BMI and stress levels were similar between groups. PAFs in former and never-smokers were similar to the whole population, whereas in current smokers the largest PAF was for stress (mean 61%, but with a wide 95% CI, − 9.3 to 86.1%), with smaller contribution from overweight and obesity, but increased contribution from respiratory disease (PAF 28.6–45.3%; Additional file 1: Table S21).

#### By presence of self-reported cardiorespiratory disease

Breathlessness was more common in people with than without self-reported cardiorespiratory disease (10.1% vs. 2.4%). Of people without self-reported cardiorespiratory disease, 13% were found to have at least one respiratory condition upon examination (CAL 8%; emphysema on HRCT 5%; and restrictive spirometry pattern 1.5%; Additional file 1: Table S25). The presence of a respiratory condition was associated with having more stress and depression (Additional file 1: Table S25), but was related to only about 4% of breathlessness cases (Additional file 1: Table S26).

## Discussion

In this large population-based study, the medical conditions most strongly related to the prevalence of breathlessness among middle-aged people were overweight and obesity (mean PAF 63%), stress (60%), respiratory disease (29%), depression (22%), and, to a smaller extent, cardiac disease (9.5%). Co-occurrence of several breathlessness-related medical conditions was common. In women and in current smokers, stress was the factor most commonly related (with the highest PAF) to the burden of breathlessness. In people without self-reported (known) cardiorespiratory disease, as many as 13% were found to have a respiratory condition upon examination—mostly airflow limitation or a restrictive spirometry pattern, which was associated with experiencing more stress and depression.

This is the first large population-based study to evaluate which medical conditions that were most strongly related to the burden of breathlessness using both self-reported, physiologic, radiologic, and blood test data. The large sample size enabled us to evaluate a large number of medical conditions in clinically relevant subgroups. This study provides novel data on the most common underlying medical conditions to consider in the clinical evaluation of people with breathlessness, and given the population-based design, the findings may pertain particularly to people evaluated in primary care. The present findings also inform on key lifestyle and public health interventions to decrease the burden of breathlessness in the community.

Middle-aged people are an important target population, as this is the age group where the prevalence of moderate to severe breathlessness (mMRC ≥ 2) starts to increase more steeply [2, 34], and as underlying conditions—if not identified and reversed/treated—can deteriorate and contribute to worse outcomes [35]. Importantly, the present findings suggest that most of the main conditions related to breathlessness in the population are amenable to health interventions for improved prevention and management—including obesity and overweight, respiratory conditions (mainly due to smoking and other noxious exposures, and asthma), cardiovascular disease (and risk factors), stress, and depression.

The key finding that the majority of breathlessness cases relate to overweight or obesity *and/or* stress suggests that the burden of breathlessness may increase substantially in coming decades due to the global trends of increasing mental health issues [36] and BMI—where the prevalence of obesity has tripled since the 1970s [37]. Importantly, this increase in breathlessness may be prevented and reversed by health interventions that target the identified main underlying conditions. The present findings supports previous reports [2, 13] that the increasing breathlessness with higher BMI is not primarily due to a higher prevalence of respiratory, cardiovascular, or other comorbidities in people with overweight/obesity, as the association was unchanged when adjusting for the presence of the other medical conditions.

Stress was one of the main factors related to the burden of breathlessness across the population and evaluated subgroups in the present study, but the association was more variable (as reflected by wider CIs) than for many of the other factors, implying that the relationship between stress and breathlessness differs more between individuals. Given the cross-sectional data, the associations do not infer direct causality, as the association for stress (and other factors such as depression) could both reflect its impact on breathlessness perception, but also, partly, reverse causality where breathlessness might worsen the factors such as stress.

Limitations of the present study include, first, the lack of some physiological assessments such as cardiac ultrasound for evaluation of heart failure. Therefore, the PAFs for some conditions may be underestimated. However, the prevalence of undiagnosed heart failure is reported to be low among middle-aged people, although most data pertain to heart failure with reduced ejection fraction [38]. In the present study, a NT-proBNP < 300 ng/ml (where heart failure is less likely) was present in 94% of all people with breathlessness, and in 97.3% of people with breathlessness without any self-reported cardiac disease. Second, lower self-reported physical activity was associated with having more breathlessness, but the influence of aerobic fitness could not be evaluated as data were lacking. Lower aerobic fitness related to worse exertional breathlessness in a previous smaller study [3]. Third, people who were excluded from the analysis due to missing data tended to have higher rates of smoking and medical conditions. Therefore, the relation between the medical conditions and breathlessness may, in fact, be underestimated. Fourth, the present findings pertain to associations and causality cannot be inferred. Fifth, breathlessness was measured using the mMRC scale [4], which is widely used in population studies and strongly associated with health outcomes [10]. As the mMRC is likely to underestimate the presence and burden of breathlessness [39], the symptom should optimally be assessed using multidimensional questionnaires [23] and at a standardized level of exertion [40–42] in future studies.

Further research is needed on the impact of medical conditions in younger and older age groups and on the broader interplay between physiological, psychological, social, and environmental factors on breathlessness, including from longitudinal studies [10].

## Conclusions

In this large population-based study of middle-aged people in Sweden, the burden of breathlessness in the population was mainly related to overweight/obesity and stress and to a lesser extent to comorbidities like respiratory, depressive, and cardiac disorders. These findings inform on key factors to consider in the clinical evaluation of people with breathlessness, and support the importance of life style interventions to prevent and reduce the burden of breathlessness across the population.

### Supplementary Information

**Additional file 1: Figure S1.** Directed Acyclical Graph (DAG) of confounding factors included in the analysis. **Table S1. **Definitions and categories of medical conditions in the analysis. **Table S2.** Characteristics and conditions in people included or excluded in the analyses. **Table S3.** Characteristics by sex. **Table S4.** Factors of interest by sex. **Table S5.** Characteristics by the presence of breathlessness in men. **Table S6.** Underlying conditions in relation to breathlessness in men. **Table S7.** Population attributable fractions of breathlessness related to underlying medical conditions in men. **Table S8.** Characteristics by the presence of breathlessness in women. **Table S9.** Underlying conditions in relation to breathlessness in women. **Table S10.** Population attributable fractions of breathlessness related to underlying medical conditions in women. **Table S11.** Characteristics by smoking history. **Table S12.** Factors of interest by smoking history. **Table S13.** Characteristics by the presence of breathlessness in neversmokers. **Table S14.** Underlying conditions in relation to breathlessness in never-smokers. **Table S15.** Population attributable fractions of breathlessness related to underlying medical conditions in never-smokers. **Table S16.** Characteristics by the presence of breathlessness in former smokers. **Table S17.** Underlying conditions in relation to breathlessness in former smokers. **Table S18.** Population attributable fractions of breathlessness related to underlying medical conditions in former smokers. **Table S19.** Characteristics by the presence of breathlessness in current smokers. **Table S20.** Underlying conditions in relation to breathlessness in current smokers. **Table S21.** Population attributable fractions of breathlessness related to underlying medical conditions in current smokers. **Table S22.** Characteristics by presence of self-reported cardiorespiratory disease. **Table S23.** Factors of interest by presence of self-reported cardiorespiratory disease. **Table S24.** Characteristics by the presence of breathlessness in people without self-reported cardiorespiratory disease. **Table S25.** Underlying conditions in relation to breathlessness in people without self-reported cardiorespiratory disease. **Table S26.** Population attributable fractions of breathlessness related to underlying medical conditions in people without self-reported cardiorespiratory disease. **Table S27.** Characteristics by the presence of breathlessness in people with self-reported cardiorespiratory disease. **Table S28.** Underlying conditions in relation to breathlessness in people with self-reported cardiorespiratory disease. **Table S29.** Population attributable fractions of breathlessness related to underlying medical conditions in people with self-reported cardiorespiratory disease.

## Data Availability

Because of the sensitive nature of the personal data and study materials, they cannot be made freely available. However, by contacting the study organization (www.scapis.org; Email: scapis@scapis.org), procedures for sharing data, analytic methods, and study materials for reproducing the results or replicating the procedure can be arranged following Swedish legislation.

## Abbreviations

aOR: Adjusted odds ratio
BMI: Body mass index
CABG: Coronary artery bypass graft
CAC: Coronary artery calcifications
CAL: Chronic airflow limitation
CCTA: Coronary computed tomography angiography
CI: Confidence interval
COPD: Chronic obstructive pulmonary disease
CT: Computed tomography
FEV_1_: Forced expiratory volume in one second
FVC: Forced vital capacity
HRCT: High-resolution computed tomography
IQR: Interquartile range
LLN: Lower limit of normal
mMRC: Modified Medical Research Council
NT-proBNP: N-terminal pro b-type natriuretic peptide
OR: Odds ratio
PAF: Population attributable fraction
PCI: Percutaneous coronary intervention
SCAPIS: Swedish CArdioPulmonary bioImage Study
