# Supplementary material for: Exertional breathlessness related to medical conditions in middle-aged people: the population-based SCAPIS study of more than 25,000 men and women
Source: Respir Res. 2024 Mar 16;25:127. doi: 10.1186/s12931-024-02766-6 (PMC10944596; doi:10.1186/s12931-024-02766-6)
Supplement: Supplementary file 1 — Additional file 1: Figure S1. Directed Acyclical Graph (DAG) of confounding factors included in the analysis. Table S1. Definitions and categories of medical conditions in the analysis. Table S2. Characteristics and conditions in people included or excluded in the analyses. Table S3. Characteristics by sex. Table S4. Factors of interest by sex. Table S5. Characteristics by the presence of breathlessness in men. Table S6. Underlying conditions in relation to breathlessness in men. Table S7. Population attributable fractions of breathlessness related to underlying medical conditions in men. Table S8. Characteristics by the presence of breathlessness in women. Table S9. Underlying conditions in relation to breathlessness in women. Table S10. Population attributable fractions of breathlessness related to underlying medical conditions in women. Table S11. Characteristics by smoking history. Table S12. Factors of interest by smoking history. Table S13. Characteristics by the presence of breathlessness in neversmokers. Table S14. Underlying conditions in relation to breathlessness in never-smokers. Table S15. Population attributable fractions of breathlessness related to underlying medical conditions in never-smokers. Table S16. Characteristics by the presence of breathlessness in former smokers. Table S17. Underlying conditions in relation to breathlessness in former smokers. Table S18. Population attributable fractions of breathlessness related to underlying medical conditions in former smokers. Table S19. Characteristics by the presence of breathlessness in current smokers. Table S20. Underlying conditions in relation to breathlessness in current smokers. Table S21. Population attributable fractions of breathlessness related to underlying medical conditions in current smokers. Table S22. Characteristics by presence of self-reported cardiorespiratory disease. Table S23. Factors of interest by presence of self-reported cardiorespiratory disease. Table S24. Characteristics [file 12931_2024_2766_MOESM1_ESM.docx]

**ADDITIONAL MATERIAL**

**Exertional breathlessness related to medical conditions in middle-aged people: the population-based SCAPIS study of more than 25,000 men and women**

**Figure S1.** Directed Acyclical Graph (DAG) of confounding factors included in the analysis

**
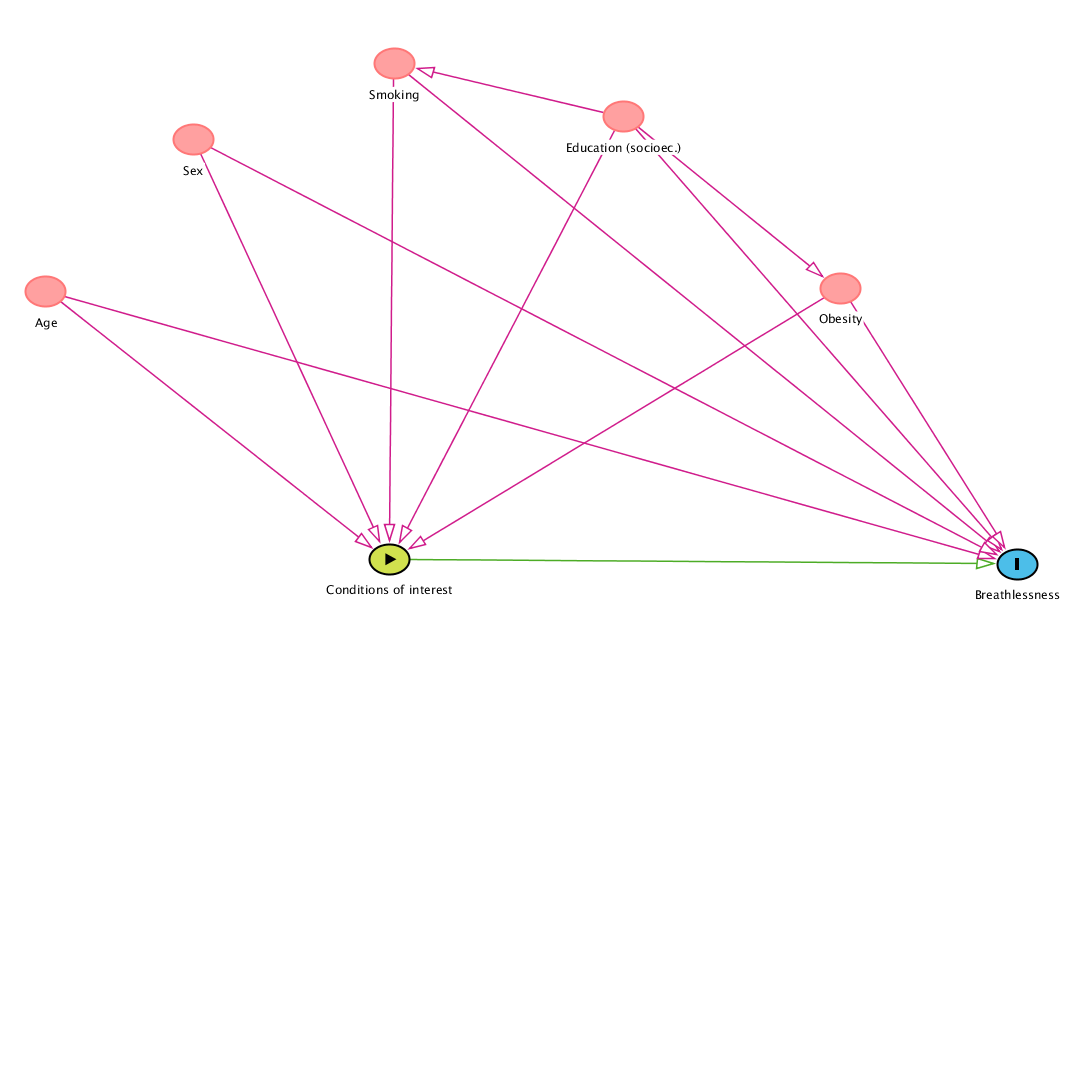
**

**Table S1.** Definitions and categories of medical conditions in the analysis

| **Condition** | **Source** | **Definition** |
| --- | --- | --- |
| *Self-reported data* |  |  |
| Asthma | Questionnaire | ‘Which of these diseases have you been diagnosed with or treated for by a physician’ (option of a multiple choice question) |
| Atrial fibrillation/flutter | Questionnaire | ‘Which of these diseases have you been diagnosed with or treated for by a physician’ (option of a multiple choice question) |
| Breathlessness | Questionnaire | mMRC ≥ 2 (‘I walk slower than people of the same age on the level because of breathlessness or have to stop for breath when walking at my own pace on the level‘, or worse) |
| Coronary artery bypass graft or percutaneous coronary intervention (CABG/PCI) | Questionnaire | ‘Which of these diseases have you been diagnosed with or treated for by a physician’ (option of a multiple choice question) |
| Cardiac valvular disease | Questionnaire | ‘Which of these diseases have you been diagnosed with or treated for by a physician’ (option of a multiple choice question) |
| Chronic bronchitis | Questionnaire | Affirmative answers on ‘Do you usually bring up phlegm or do you have phlegm in your chest that is difficult to bring up, when you don't have a cold?’, and ‘Do you bring up phlegm or do you have problems with phlegm in your chest in that way on most days for at least 3 months each year?’; and ‘How many years have you had these problems with phlegm?’ selecting more than two years. |
| Chronic rhinosinusitis | Questionnaire | At least two symptoms, of which one must be a major symptom (nasal obstruction or nasal discharge) for more than 12 weeks. Major symptom: · Have you had a blocked nose for more than 12 weeks in the last 12 month · Have you experienced discolored mucus in your throat for more than 12 weeks in the last 12 month Minor symptom: · Have you experienced pain or pressure around your forehead, nose, or eyes for more than 12 weeks in the last 12 month? · Has your sense of smell been reduced or absent for more than 12 weeks in the last 12 months? |
| Depression | Questionnaire | Affirmative answer to ‘During the past twelve months, was there ever a time when you felt sad, blue, or depressed for two weeks or more in a row?’, and affirmed at least five of the seven related questions of having [[1](#_ENREF_1)]:  1. ‘Lost interest in most things like hobbies, work or activities that usually give you pleasure?’  2. ‘Felt tired or low on energy?’  3. ‘Gained or lost weight?’  4. ‘Trouble falling asleep?’  5. ‘Concentration problems?’  6. ‘Thoughts about death?’  7. ‘Bad self-esteem/feeling worthless?’ |
| Heart failure | Questionnaire | ‘Which of these diseases have you been diagnosed with or treated for by a physician’ (option of a multiple choice question) |
| Ischemic heart disease | Questionnaire | Myocardial infarction or CABG/PCI as response to: ‘Which of these diseases have you been diagnosed with or treated for by a physician’ (option of a multiple choice question) |
| Myocardial infarction | Questionnaire | ‘Which of these diseases have you been diagnosed with or treated for by a physician’ (option of a multiple choice question) |
| Other respiratory disease | Questionnaire | ‘Which of these diseases have you been diagnosed with or treated for by a physician’ (option of a multiple choice question) |
| Peripheral arterial insufficiency | Questionnaire | ‘Which of these diseases have you been diagnosed with or treated for by a physician’ (option of a multiple choice question) |
| Physical exercise level | Questionnaire | Categorized using four questions adopted from the Public Health Agency of Sweden [[2](#_ENREF_2)] as: high (regular hard exercise >30 min, > 3 times/week), medium (exercise such as walking or cycling for at least 4 hours per week) or low (spending most leisure time at sedentary activities with light physical exercise <2 hours per week) level of physical exercise. |
| Stress level | Questionnaire | Assessed using the question: “By stress we mean feeling tense, irritable, anxious or having sleeping difficulties as a result of conditions at work or at home. Did you experience this?”. It was self-rated on a 5-point ordinal scale in accordance with Rosengren *et al.* [[1](#_ENREF_1), [3](#_ENREF_3)]: 0 “never”, 1 “any stress period”, 2 “some stress periods during the last five years”, 3 “constant stress during the last year”, and 4 “constant stress during the last five years”. In the analyses, the presence of stress was categorized as a score of ≥ 2. |
| *Measured data* |  |  |
| Anemia | Blood test | Venous haemoglobin (Hb) < 120 g/L in women and < 130 g/L in men, according to the World Health Organization classification [[4](#_ENREF_4)]. |
| Chronic airflow limitation (CAL) | Spirometry | Forced expired volume in one second / forced vital capacity (FEV_1_/FVC) < 0.7 post-bronchodilation. |
| Coronary artery calcification (CAC) total score | Cardiac noncontrast computed tomography (CT) | Assessed using non-contrast computed tomography CT, as previously described [21]. CAC total score (in Agatston Units) were categorized as 0 (none), 1 –100 (low), or >100 (moderate to high) [21]. |
| Coronary artery stenosis | Cardiac CT angiography | Assessed using coronary computed tomography angiography (CCTA), as detailed elsewhere [21], in accordance with the American College of Cardiology Foundation/American Heart Association Clinical Competence Statement on cardiac CT [26]. |
| Pulmonary emphysema (HRCT) |  | Assessed through high-resolution computed tomography (HRCT), using a Somatom Definition Flash scanner (Siemens Healthcare, Forchheim, Germany). The methodology has been described in detail [26, 27]. CT scanners at each of the six sites used identical software, exam protocols and hardware throughout the study, and the CT scans were read by trained readers at each site.  Emphysema was graded as none (0), mild (1), moderate (2) or severe (3) together with localisation in the upper, middle and/or lower part of right and/or left lung. The imaging terminology was based on suggested terminology by the Fleischner Society [28]. Presence of emphysema was defined as at least mild emphysema (grade 1) in any location, in accordance with the validation of the visual assessment of mild emphysema in the SCAPIS pilot study [27]. |
| Obesity | Examination | Measured body mass index (BMI) ≥ 30 kg/m^2^. |
| Overweight | Examination | BMI 25-29.9 kg/m^2^. |
| Restrictive spirometry pattern | Spirometry | FVC < LLN and FEV_1_/FVC > LLN post-bronchodilation |

*Abbreviations:* CABG, coronary artery bypass graft surgery; CAC, coronary artery calcifications; CT, computed tomography; FEV_1_, forced expired volume in one second; FVC, forced vital capacity; LLN, lower limit of normal; HRCT, high resolution computed tomography; mMRC, modified Medical Research Council breathlessness score; PCI, percutaneous coronary intervention.

**Table S2.** Characteristics and conditions in people included or excluded in the analyses

|  | **Included** | **Excluded** |
| --- | --- | --- |
|  | N=25,948 (86%) | N=4,206 (14%) |
| Age (years) | 57.5 (4.4) | 57.7 (4.3) |
| Female sex | 13,265 (51.1%) | 2,243 (53.3%) |
| mMRC breathlessness rating |  |  |
| 0 | 23,741 (91.5%) | 2,354 (56.0%) |
| 1 | 1,255 (4.8%) | 288 (6.8%) |
| 2 | 773 (3.0%) | 231 (5.5%) |
| 3 | 88 (0.3%) | 64 (1.5%) |
| 4 | 91 (0.4%) | 84 (2.0%) |
| Smoking history | 0 (0.0%) | 1,185 (28.2%) |
| Never | 13,348 (51.4%) | 1,384 (32.9%) |
| Former | 9,506 (36.6%) | 1,096 (26.1%) |
| Current | 3,094 (11.9%) | 764 (18.2%) |
| Missing | 0 (0.0%) | 962 (22.9%) |
| Pack-years of smoking | 7.4 (11.8) | 11.5 (14.9) |
| Body mass index (kg/m^2^) |  |  |
| <25.0 | 9,383 (36.2%) | 1,248 (29.7%) |
| 25-29.9 | 11,250 (43.4%) | 1,786 (42.5%) |
| ≥30 | 5,315 (20.5%) | 1,168 (27.8%) |
| Missing | 0 (0.0%) | 4 (0.1%) |
| FEV_1_ (l) | 3.3 (0.8) | 3.1 (0.8) |
| FEV_1_ (%pred) | 102.5 (13.7) | 99.3 (15.4) |
| FVC (l) | 4.2 (1.0) | 4.0 (1.0) |
| FVC (%pred) | 103.0 (12.8) | 100.2 (14.0) |
| FEV_1_/FVC | 0.8 (0.1) | 0.8 (0.1) |
| FEV_1_/FVC (%pred) | 99.2 (7.9) | 98.7 (9.0) |
| Highest completed education |  |  |
| University | 11,968 (46.1%) | 1,250 (29.7%) |
| Secondary | 11,693 (45.1%) | 1,642 (39.0%) |
| Primary or none | 2,231 (8.6%) | 516 (12.3%) |
| Missing | 56 (0.2%) | 798 (19.0%) |
| Residence |  |  |
| Own house | 12,434 (47.9%) | 1,243 (29.6%) |
| Own apartment | 7,724 (29.8%) | 988 (23.5%) |
| Rented apartment | 5,586 (21.5%) | 1,105 (26.3%) |
| Other | 188 (0.7%) | 56 (1.3%) |
| Missing | 16 (0.1%) | 814 (19.4%) |
| Respiratory disease | 6,464 (24.9%) | 1,209 (28.7%) |
| Asthma | 2,025 (7.8%) | 397 (10.4%) |
| Chronic airflow limitation | 2,706 (10.4%) | 466 (12.3%) |
| Emphysema (HRCT) | 1,427 (5.5%) | 261 (7.2%) |
| Chronic bronchitis | 707 (2.7%) | 194 (6.5%) |
| Chronic rhinosinusitis | 564 (2.2%) | 115 (4.1%) |
| Restrictive spirometry pattern | 466 (1.8%) | 145 (3.8%) |
| Other respiratory disease | 320 (1.2%) | 79 (2.6%) |
| Body mass index (kg/m2) |  |  |
| <25.0 | 9,383 (36.2%) | 1,248 (29.7%) |
| 25-29.9 | 11,250 (43.4%) | 1,786 (42.5%) |
| ≥30 | 5,315 (20.5%) | 1,168 (27.8%) |
| Missing | 0 (0.0%) | 4 (0.1%) |
| Anemia | 886 (3.4%) | 212 (5.3%) |
| Peripheral arterial insufficiency | 73 (0.3%) | 17 (0.5%) |
| Stress level |  |  |
| Never stress | 1,285 (5.0%) | 143 (3.4%) |
| Any stress period | 9,261 (35.7%) | 959 (22.8%) |
| Some stress periods (last 5 yrs) | 10,082 (38.9%) | 968 (23.0%) |
| Constant stress (last 1 year) | 2,625 (10.1%) | 398 (9.5%) |
| Constant stress (last 5 yrs) | 2,695 (10.4%) | 499 (11.9%) |
| Missing | 0 (0.0%) | 1,239 (29.5%) |
| Depression | 3,700 (14.3%) | 640 (23.0%) |
| Cardiac disease | 2,382 (9.2%) | 399 (10.0%) |
| Coronary artery stenosis | 1,377 (5.7%) | 227 (7.2%) |
| Ischemic heart disease | 500 (1.9%) | 98 (3.2%) |
| Myocardial infarction | 396 (1.5%) | 77 (2.5%) |
| Prior CABG/PCI | 266 (1.0%) | 43 (1.4%) |
| Atrial fibrillation/flutter | 458 (1.8%) | 80 (2.6%) |
| Cardiac valvular disease | 207 (0.8%) | 43 (1.4%) |
| Heart failure | 119 (0.5%) | 30 (1.0%) |
| CAC total score, median (IQR) | 0.0 (0.0-19.0) | 0.0 (0.0-33.0) |
| CAC category |  |  |
| 0 | 15,127 (58.3%) | 1,964 (46.7%) |
| 1-100 | 7,261 (28.0%) | 1,028 (24.4%) |
| >100 | 3,003 (11.6%) | 547 (13.0%) |
| Missing | 557 (2.1%) | 667 (15.9%) |

Data are presented as mean (standard deviation) or frequency (%).

*Abbreviations:* BMI, body mass index; COPD, chronic obstructive pulmonary disease; FEV_1_, forced expiratory volume in 1 second; FVC, forced vital capacity; mMRC, modified Medical Research Council breathlessness score; pred, predicted value.

**Subgroup analysis by: SEX**

**Table S3.** Characteristics by sex

|  | **Men** | **Women** |
| --- | --- | --- |
|  | N=12,683 | N=13,265 |
| Age (years) | 57.5 (4.4) | 57.5 (4.3) |
| Female sex | 0 (0.0%) | 13,265 (100.0%) |
| Smoking history |  |  |
| Never | 6,927 (54.6%) | 6,421 (48.4%) |
| Former | 4,248 (33.5%) | 5,258 (39.6%) |
| Current | 1,508 (11.9%) | 1,586 (12.0%) |
| Pack-years of smoking | 7.4 (12.5) | 7.4 (11.1) |
| Body mass index (kg/m2) |  |  |
| <25.0 | 3,478 (27.4%) | 5,905 (44.5%) |
| 25-29.9 | 6,471 (51.0%) | 4,779 (36.0%) |
| ≥30 | 2,734 (21.6%) | 2,581 (19.5%) |
| FEV_1_ (l) | 3.8 (0.6) | 2.8 (0.4) |
| FEV_1_ (%pred) | 101.7 (13.7) | 103.2 (13.6) |
| FVC (l) | 5.0 (0.8) | 3.5 (0.6) |
| FVC (%pred) | 101.9 (12.7) | 104.1 (12.8) |
| FEV_1_/FVC | 0.8 (0.1) | 0.8 (0.1) |
| FEV_1_/FVC (%pred) | 99.7 (8.2) | 98.7 (7.4) |
| Highest completed education |  |  |
| University | 5,297 (41.8%) | 6,671 (50.3%) |
| Secondary | 6,111 (48.2%) | 5,582 (42.1%) |
| Primary or none | 1,248 (9.8%) | 983 (7.4%) |
| Missing | 27 (0.2%) | 29 (0.2%) |
| Residence |  |  |
| Own house | 6,434 (50.7%) | 6,000 (45.2%) |
| Own apartment | 3,553 (28.0%) | 4,171 (31.4%) |
| Rented apartment | 2,588 (20.4%) | 2,998 (22.6%) |
| Other | 100 (0.8%) | 88 (0.7%) |
| Missing | 8 (0.1%) | 8 (0.1%) |

*Abbreviations:* please see Table S1.

**Table S4.** Factors of interest by sex

|  | **Men** | **Women** |
| --- | --- | --- |
|  | N=12,683 | N=13,265 |
| Respiratory disease | 3,293 (26.0%) | 3,171 (23.9%) |
| Asthma | 827 (6.5%) | 1,198 (9.0%) |
| Chronic airflow limitation | 1,559 (12.3%) | 1,147 (8.6%) |
| Emphysema (HRCT) | 759 (6.0%) | 668 (5.0%) |
| Chronic bronchitis | 376 (3.0%) | 331 (2.5%) |
| Chronic rhinosinuitis | 239 (1.9%) | 325 (2.5%) |
| Restrictive spirometry pattern | 275 (2.2%) | 191 (1.4%) |
| Other respiratory disease | 149 (1.2%) | 171 (1.3%) |
| Body mass index (kg/m^2^) |  |  |
| <25.0 | 3,478 (27.4%) | 5,905 (44.5%) |
| 25-29.9 | 6,471 (51.0%) | 4,779 (36.0%) |
| ≥30 | 2,734 (21.6%) | 2,581 (19.5%) |
| Anemia | 282 (2.2%) | 604 (4.6%) |
| Peripheral arterial insufficiency | 48 (0.4%) | 25 (0.2%) |
| Stress level |  |  |
| Never stress | 900 (7.1%) | 385 (2.9%) |
| Any stress period | 4,872 (38.4%) | 4,389 (33.1%) |
| Some stress periods (last 5 yrs) | 4,891 (38.6%) | 5,191 (39.1%) |
| Constant stress (last 1 year) | 996 (7.9%) | 1,629 (12.3%) |
| Constant stress (last 5 yrs) | 1,024 (8.1%) | 1,671 (12.6%) |
| Depression | 1,154 (9.1%) | 2,546 (19.2%) |
| Cardiac disease | 1,781 (14.0%) | 601 (4.5%) |
| Coronary artery stenosis | 1,083 (9.0%) | 294 (2.4%) |
| Ischemic heart disease | 389 (3.1%) | 111 (0.8%) |
| Myocardial infarction | 304 (2.4%) | 92 (0.7%) |
| Prior CABG/PCI | 222 (1.8%) | 44 (0.3%) |
| Atrial fibrillation/flutter | 347 (2.7%) | 111 (0.8%) |
| Cardiac valvular disease | 114 (0.9%) | 93 (0.7%) |
| Heart failure | 81 (0.6%) | 38 (0.3%) |
| CAC total score | 2.0 (0.0-55.0) | 0.0 (0.0-2.0) |
| CAC category |  |  |
| 0 | 5,633 (44.4%) | 9,494 (71.6%) |
| 1-100 | 4,379 (34.5%) | 2,882 (21.7%) |
| >100 | 2,263 (17.8%) | 740 (5.6%) |
| Missing | 408 (3.2%) | 149 (1.1%) |

*Abbreviations:* please see Table S1.

**MEN**

**Table S5.** Characteristics by the presence of breathlessness in men

|  | **Without breathlessness**  **(mMRC 0-1)** | **With breathlessness (mMRC ≥ 2)** |
| --- | --- | --- |
|  | N=12,377 | N=306 |
| Age (years) | 57.5 (4.4) | 58.3 (4.3) |
| Female sex | 0 (0.0%) | 0 (0.0%) |
| Smoking history |  |  |
| Never | 6,816 (55.1%) | 111 (36.3%) |
| Former | 4,127 (33.3%) | 121 (39.5%) |
| Current | 1,434 (11.6%) | 74 (24.2%) |
| Pack-years of smoking | 7.2 (12.1) | 17.3 (19.8) |
| Body mass index (kg/m^2^) |  |  |
| <25.0 | 3,450 (27.9%) | 28 (9.2%) |
| 25-29.9 | 6,366 (51.4%) | 105 (34.3%) |
| ≥30 | 2,561 (20.7%) | 173 (56.5%) |
| FEV_1_ (l) | 3.8 (0.6) | 3.2 (0.8) |
| FEV_1_ (%pred) | 102.0 (13.4) | 88.2 (18.1) |
| FVC (l) | 5.0 (0.8) | 4.3 (0.8) |
| FVC (%pred) | 102.1 (12.6) | 92.7 (14.2) |
| FEV_1_/FVC | 0.8 (0.1) | 0.7 (0.1) |
| FEV_1_/FVC (%pred) | 99.8 (8.0) | 94.8 (12.9) |
| Highest completed education |  |  |
| University | 5,234 (42.3%) | 63 (20.6%) |
| Secondary | 5,934 (47.9%) | 177 (57.8%) |
| Primary or none | 1,183 (9.6%) | 65 (21.2%) |
| Missing | 26 (0.2%) | 1 (0.3%) |
| Residence |  |  |
| Own house | 6,325 (51.1%) | 109 (35.6%) |
| Own apartment | 3,479 (28.1%) | 74 (24.2%) |
| Rented apartment | 2,469 (19.9%) | 119 (38.9%) |
| Other | 96 (0.8%) | 4 (1.3%) |
| Missing | 8 (0.1%) | 0 (0.0%) |

Data are presented as mean (standard deviation) or frequency (%). *Abbreviations:* please see Table S1.

**Table S6.** Underlying conditions in relation to breathlessness in men

|  | **Without breathlessness**  **(mMRC 0-1)** | **With breathlessness**  **(mMRC ≥ 2)** | **Association with breathlessness**  Odds ratio (95% CI) | |
| --- | --- | --- | --- | --- |
|  |  |  | **Crude** | **Adjusted for confounders*** |
| Respiratory disease | 3,134 (25.3%) | 159 (52.0%) | 3.2 (2.2-4.7) | 2.6 (1.7-3.9) |
| Asthma | 794 (6.4%) | 33 (10.8%) | 1.8 (1.4-2.2) | 1.8 (1.5-2.3) |
| Chronic airflow limitation | 1,473 (11.9%) | 86 (28.1%) | 2.9 (2.2-3.7) | 2.5 (2.0-3.3) |
| Emphysema (HRCT) | 710 (5.7%) | 49 (16.0%) | 3.1 (1.9-5.1) | 2.1 (1.3-3.7) |
| Chronic bronchitis | 336 (2.7%) | 40 (13.1%) | 5.4 (3.4-8.6) | 3.8 (2.3-6.3) |
| Chronic rhinosinuitis | 230 (1.9%) | 9 (2.9%) | 1.6 (0.8-3.1) | 1.5 (0.8-2.8) |
| Restrictive spirometry pattern | 247 (2.0%) | 28 (9.2%) | 4.9 (3.1-7.9) | 3.1 (1.9- 5.0) |
| Other respiratory disease | 136 (1.1%) | 13 (4.2%) | 4.0 (2.1-7.5) | 4.3 (2.3-8.3) |
| Body mass index (kg/m^2^) |  |  |  |  |
| <25.0 | 3,450 (27.9%) | 28 (9.2%) | 1 (Ref) | 1 (Ref) |
| 25-29.9 | 6,366 (51.4%) | 105 (34.3%) | 2.0 (1.3-3.1) | 2.0 (1.3-2.9) |
| ≥30 | 2,561 (20.7%) | 173 (56.5%) | 8.3 (5.9-11.8) | 7.1 (5.6-9.1) |
| Anemia | 269 (2.2%) | 13 (4.2%) | 2.0 (1.4-2.9) | 1.8 (1.2-2.5) |
| Peripheral arterial insufficiency | 43 (0.3%) | 5 (1.6%) | 4.8 (1.7-13.2) | 3.2 (1.4-7.6) |
| Stress level |  |  |  |  |
| Never stress | 886 (7.2%) | 14 (4.6%) | 1 (Ref) | 1 (Ref) |
| Any stress period | 4,786 (38.7%) | 86 (28.1%) | 1.1 (0.5-2.5) | 1.6 (0.7-3.6) |
| Some stress periods (last 5 yrs) | 4,794 (38.7%) | 97 (31.7%) | 1.3 (0.7-2.4) | 2.0 (1.0-3.7) |
| Constant stress (last 1 year) | 959 (7.7%) | 37 (12.1%) | 2.4 (1.2-4.8) | 3.5 (1.7-7.2) |
| Constant stress (last 5 yrs) | 952 (7.7%) | 72 (23.5%) | 4.8 (2.7-8.6) | 6.7 (3.8-11.6) |
| Depression | 1,064 (8.6%) | 90 (29.4%) | 4.4 (3.2-6.1) | 3.8 (2.7-5.4) |
| Cardiac disease | 1,687 (13.6%) | 94 (30.7%) | 2.8 (2.4-3.3) | 2.1 (1.8-2.6) |
| Coronary artery stenosis | 1,038 (8.8%) | 45 (16.9%) | 2.1 (1.8-2.5) | 1.5 (1.3-1.8) |
| Ischemic heart disease | 359 (2.9%) | 30 (9.8%) | 3.6 (2.9-4.6) | 2.2 (1.6-3.0) |
| Myocardial infarction | 279 (2.3%) | 25 (8.2%) | 3.9 (2.8-5.3) | 2.3 (1.6-3.3) |
| CABG/PCI | 207 (1.7%) | 15 (4.9%) | 3.0 (2.0-4.7) | 1.9 (1.2-3.1) |
| Atrial fibrillation/flutter | 323 (2.6%) | 24 (7.8%) | 3.2 (1.9-5.2) | 2.8 (1.8-4.6) |
| Cardiac valvular disease | 108 (0.9%) | 6 (2.0%) | 2.3 (0.7-7.4) | 2.4 (0.6-9.4) |
| Heart failure | 63 (0.5%) | 18 (5.9%) | 12.2 (7.0-21.3) | 9.8 (5.4-17.9) |
| CAC total score | 2.0 (0.0-54.0) | 18.0 (0.0-159.0) | - | - |
| CAC category |  |  |  |  |
| 0 | 5,543 (44.8%) | 90 (29.4%) | 1 (Ref) | 1 (Ref) |
| 1-100 | 4,274 (34.5%) | 105 (34.3%) | 1.5 (1.2-1.9) | 1.2 (0.9-1.5) |
| >100 | 2,175 (17.6%) | 88 (28.8%) | 2.5 (1.9-3.2) | 1.5 (1.2-1.9) |
| Missing | 385 (3.1%) | 23 (7.5%) | - | - |

Data presented as frequency (%) and associations as odds ratio (95% confidence interval) analyzed using logistic regression.

^*^Adjusted for age, sex, smoking history, pack-years of smoking, highest completed education, and body mass index (when appropriate).

*Abbreviations:* please see Table S1.

**Table S7.** Population attributable fractions of breathlessness related to underlying medical conditions in men

| **Condition** | **PAF (95% CI)** |
| --- | --- |
| Overweight and obesity | 0.644 (0.539-0.725) |
| Stress | 0.523 (0.134-0.738) |
| Respiratory disease | 0.306 (0.156-0.429) |
| Asthma | 0.045 (0.024-0.066) |
| CAL | 0.160 (0.101-0.215) |
| Emphysema (on HRCT) | 0.078 (0.009-0.143) |
| Chronic bronchitis | 0.090 (0.041-0.136) |
| Chronic rhinosinuitis | 0.009 (0.000-0.026) |
| Restrictive spirometry pattern | 0.057 (0.023-0.090) |
| Other respiratory disease | 0.031 (0.009-0.052) |
| Depression | 0.204 (0.130-0.271) |
| Cardiac disease | 0.153 (0.111-0.193) |
| Coronary artery stenosis | 0.053 (0.029-0.077) |
| Heart failure | 0.050 (0.029-0.070) |
| Ischemic heart disease | 0.049 (0.026-0.072) |
| Myocardial infarction | 0.041 (0.018-0.064) |
| CABG/PCI | 0.021 (0.002-0.040) |
| Atrial fibrillation/flutter | 0.048 (0.017-0.079) |
| Cardiac valvular disease | 0.011 (-0.013-0.034) |
| CAC | 0.155 (0.051-0.248) |
| Anemia | 0.016 (0.004-0.028) |
| Peripheral arterial insufficiency | 0.010 (0.000-0.022) |

For explanations and abbreviations, see Table 3 in the main article.

*Adjusted for age, sex, smoking history, pack-years, highest education and BMI (except when BMI is analyzed as factor).

**WOMEN**

**Table S8.** Characteristics by the presence of breathlessness in women

|  | **Without breathlessness**  **(mMRC 0-1)** | **With breathlessness (mMRC ≥ 2)** |
| --- | --- | --- |
|  | N=12,619 | N=646 |
| Age (years) | 57.4 (4.3) | 58.3 (4.5) |
| Female sex | 12,619 (100.0%) | 646 (100.0%) |
| Smoking history |  |  |
| Never | 6,171 (48.9%) | 250 (38.7%) |
| Former | 4,965 (39.3%) | 293 (45.4%) |
| Current | 1,483 (11.8%) | 103 (15.9%) |
| Pack-years of smoking | 7.1 (10.7) | 12.7 (15.7) |
| Body mass index (kg/m^2^) |  |  |
| <25.0 | 5,805 (46.0%) | 100 (15.5%) |
| 25-29.9 | 4,591 (36.4%) | 188 (29.1%) |
| ≥30 | 2,223 (17.6%) | 358 (55.4%) |
| FEV_1_ (l) | 2.8 (0.4) | 2.5 (0.5) |
| FEV_1_ (%pred) | 103.7 (13.3) | 94.6 (16.1) |
| FVC (l) | 3.6 (0.6) | 3.2 (0.6) |
| FVC (%pred) | 104.4 (12.7) | 97.7 (13.5) |
| FEV_1_/FVC | 0.8 (0.1) | 0.8 (0.1) |
| FEV_1_/FVC (%pred) | 98.8 (7.2) | 96.3 (10.9) |
| Highest completed education |  |  |
| University | 6,458 (51.2%) | 213 (33.0%) |
| Secondary | 5,265 (41.7%) | 317 (49.1%) |
| Primary or none | 868 (6.9%) | 115 (17.8%) |
| Missing | 28 (0.2%) | 1 (0.2%) |
| Residence |  |  |
| Own house | 5,780 (45.8%) | 220 (34.1%) |
| Own apartment | 3,968 (31.4%) | 203 (31.4%) |
| Rented apartment | 2,783 (22.1%) | 215 (33.3%) |
| Other | 80 (0.6%) | 8 (1.2%) |
| Missing | 8 (0.1%) | 0 (0.0%) |

Data are presented as mean (standard deviation) or frequency (%). *Abbreviations:* please see Table S1.

**Table S9.** Underlying conditions in relation to breathlessness in women

|  | **Without breathlessness**  **(mMRC 0-1)** | **With breathlessness**  **(mMRC ≥ 2)** | **Association with breathlessness**  Odds ratio (95% CI) | |
| --- | --- | --- | --- | --- |
|  |  |  | **Crude** | **Adjusted for confounders*** |
| Respiratory disease | 2,860 (22.7%) | 311 (48.1%) | 3.2 (2.6-3.8) | 2.8 (2.2-3.6) |
| Asthma | 1,042 (8.3%) | 156 (24.1%) | 3.5 (2.8-4.4) | 3.4 (2.7-4.3) |
| Chronic airflow limitation | 1,034 (8.2%) | 113 (17.5%) | 2.4 (2.0- 2.8) | 2.1 (1.7-2.6) |
| Emphysema (HRCT) | 604 (4.8%) | 64 (9.9%) | 2.2 (1.7-2.9) | 1.8 (1.2-2.6) |
| Chronic bronchitis | 268 (2.1%) | 63 (9.8%) | 5.0 (4.1-6.1) | 4.4 (3.6-5.3) |
| Chronic rhinosinuitis | 283 (2.2%) | 42 (6.5%) | 3.0 (1.6-5.6) | 2.6 (1.2-5.9) |
| Restrictive spirometry pattern | 168 (1.3%) | 23 (3.6%) | 2.7 (1.9-3.9) | 2.2 (1.6-3.1) |
| Other respiratory disease | 141 (1.1%) | 30 (4.6%) | 4.3 (3.0-6.1) | 4.2 (3.0-5.7) |
| Body mass index (kg/m^2^) |  |  |  |  |
| <25.0 | 5,805 (46.0%) | 100 (15.5%) | 1 (Ref) | 1 (Ref) |
| 25-29.9 | 4,591 (36.4%) | 188 (29.1%) | 2.4 (1.8-3.1) | 2.1 (1.6-2.8) |
| ≥30 | 2,223 (17.6%) | 358 (55.4%) | 9.3 (8.3-10.5) | 7.8 (6.8-9.0) |
| Anemia | 568 (4.5%) | 36 (5.6%) | 1.3 (0.8-1.9) | 1.8 (1.2-2.7) |
| Peripheral arterial insufficiency | 22 (0.2%) | 3 (0.5%) | 2.7 (0.9-8.3) | 2.5 (0.8-8.5) |
| Stress level |  |  |  |  |
| Never stress | 377 (3.0%) | 8 (1.2%) | 1 (Ref) | 1 (Ref) |
| Any stress period | 4,220 (33.4%) | 169 (26.2%) | 1.9 (0.8-4.4) | 2.5 (1.1-5.9) |
| Some stress periods (last 5 yrs) | 5,005 (39.7%) | 186 (28.8%) | 1.8 (0.8-3.6) | 2.7 (1.2-5.9) |
| Constant stress (last 1 year) | 1,520 (12.0%) | 109 (16.9%) | 3.4 (1.6-7.2) | 5.1 (2.3-11.4) |
| Constant stress (last 5 yrs) | 1,497 (11.9%) | 174 (26.9%) | 5.5 (2.4-12.6) | 7.9 (3.3-18.6) |
| Depression | 2,286 (18.1%) | 260 (40.2%) | 3.0 (2.6-3.6) | 2.6 (2.2-3.1) |
| Cardiac disease | 517 (4.1%) | 84 (13.0%) | 3.5 (2.6-4.7) | 2.5 (1.8-3.5) |
| Coronary artery stenosis | 263 (2.3%) | 31 (5.6%) | 2.6 (2.0-3.3) | 1.8 (1.3-2.5) |
| Ischemic heart disease | 88 (0.7%) | 23 (3.6%) | 5.3 (3.9-7.1) | 2.6 (1.5-4.4) |
| Myocardial infarction | 72 (0.6%) | 20 (3.1%) | 5.6 (4.0-7.8) | 2.6 (1.4-4.6) |
| CABG/PCI | 35 (0.3%) | 9 (1.4%) | 5.1 (2.7-9.6) | 2.4 (1.0-5.9) |
| Atrial fibrillation/flutter | 94 (0.7%) | 17 (2.6%) | 3.6 (2.7-4.9) | 2.4 (1.5-3.8) |
| Cardiac valvular disease | 76 (0.6%) | 17 (2.6%) | 4.5 (2.0-9.9) | 5.3 (2.3-11.9) |
| Heart failure | 30 (0.2%) | 8 (1.2%) | 5.3 (2.2-12.3) | 2.9 (1.2-6.9) |
| CAC total score | 0.0 (0.0-1.0) | 0.0 (0.0-11.0) | - | - |
| CAC category |  |  |  |  |
| 0 | 9,095 (72.1%) | 399 (61.8%) | 1 (Ref) | 1 (Ref) |
| 1-100 | 2,720 (21.6%) | 162 (25.1%) | 1.4 (1.2-1.5) | 1.0 (0.9-1.1) |
| >100 | 676 (5.4%) | 64 (9.9%) | 2.2 (1.7-2.7) | 1.2 (1.1-1.4) |
| Missing | 128 (1.0%) | 21 (3.3%) | - | - |

Data presented as frequency (%) and associations as odds ratio (95% confidence interval) analyzed using logistic regression.

^*^Adjusted for age, sex, smoking history, pack-years of smoking, highest completed education, and body mass index (when appropriate).

*Abbreviations:* please see Table S1.

**Table S10.** Population attributable fractions of breathlessness related to underlying medical conditions in women

| **Condition** | **PAF (95% CI)** |
| --- | --- |
| Stress | 0.684 (0.328-0.852) |
| Overweight and obesity | 0.621 (0.563-0.672) |
| Respiratory disease | 0.284 (0.209-0.352) |
| Asthma | 0.155 (0.115-0.192) |
| CAL | 0.081 (0.052-0.109) |
| Chronic bronchitis | 0.066 (0.053-0.079) |
| Emphysema (on HRCT) | 0.037 (0.009-0.065) |
| Chronic rhinosinuitis | 0.036 (-0.004-0.074) |
| Restrictive spirometry pattern | 0.017 (0.008-0.027) |
| Other respiratory disease | 0.031 (0.021-0.041) |
| Depression | 0.226 (0.185-0.266) |
| Cardiac disease | 0.068 (0.035-0.099) |
| Coronary artery stenosis | 0.021 (0.006-0.036) |
| Cardiac valvular disease | 0.020 (0.005-0.035) |
| Ischemic heart disease | 0.017 (0.005-0.029) |
| Myocardial infarction | 0.015 (0.003-0.026) |
| CABG/PCI | 0.007 (-0.001-0.015) |
| Atrial fibrillation/flutter | 0.013 (0.004-0.022) |
| Heart failure | 0.007 (0.000-0.014) |
| Anemia | 0.023 (0.004-0.041) |
| CAC | 0.012 (-0.015-0.038) |
| Peripheral arterial insufficiency | 0.003 (-0.002-0.007) |

For explanations and abbreviations, see Table 3 in the main article.

*Adjusted for age, sex, smoking history, pack-years, highest education and BMI (except when BMI is analyzed as factor).

**Subgroup analysis by: SMOKING HISTORY**

**Table S11.** Characteristics by smoking history

|  | **Never** | **Former** | **Current** |
| --- | --- | --- | --- |
|  | N=13,348 | N=9,506 | N=3,094 |
| Age (years) | 56.9 (4.3) | 58.3 (4.3) | 57.3 (4.2) |
| Female sex | 6,421 (48.1%) | 5,258 (55.3%) | 1,586 (51.3%) |
| Smoking history |  |  |  |
| Never | 13,348 (100.0%) | 0 (0.0%) | 0 (0.0%) |
| Former | 0 (0.0%) | 9,506 (100.0%) | 0 (0.0%) |
| Current | 0 (0.0%) | 0 (0.0%) | 3,094 (100.0%) |
| Pack-years of smoking | 0.0 (0.0) | 13.0 (11.4) | 22.3 (14.4) |
| Body mass index (kg/m2) |  |  |  |
| <25.0 | 5,178 (38.8%) | 3,099 (32.6%) | 1,106 (35.7%) |
| 25-29.9 | 5,732 (42.9%) | 4,189 (44.1%) | 1,329 (43.0%) |
| ≥30 | 2,438 (18.3%) | 2,218 (23.3%) | 659 (21.3%) |
| FEV1 (l) | 3.4 (0.8) | 3.2 (0.7) | 3.1 (0.8) |
| FEV1 (%pred) | 103.4 (12.9) | 102.6 (13.8) | 98.0 (15.4) |
| FVC (l) | 4.3 (1.0) | 4.1 (0.9) | 4.2 (1.0) |
| FVC (%pred) | 102.8 (12.6) | 103.5 (12.8) | 102.1 (13.4) |
| FEV1/FVC | 0.8 (0.1) | 0.8 (0.1) | 0.8 (0.1) |
| FEV1/FVC (%pred) | 100.3 (7.0) | 98.7 (7.9) | 95.7 (9.9) |
| Highest completed education |  |  |  |
| University | 7,096 (53.2%) | 3,933 (41.4%) | 939 (30.3%) |
| Secondary | 5,499 (41.2%) | 4,550 (47.9%) | 1,644 (53.1%) |
| Primary or none | 730 (5.5%) | 1,002 (10.5%) | 499 (16.1%) |
| Missing | 23 (0.2%) | 21 (0.2%) | 12 (0.4%) |
| Residence |  |  |  |
| Own house | 7,294 (54.6%) | 4,156 (43.7%) | 984 (31.8%) |
| Own apartment | 3,771 (28.3%) | 3,031 (31.9%) | 922 (29.8%) |
| Rented apartment | 2,197 (16.5%) | 2,241 (23.6%) | 1,148 (37.1%) |
| Other | 78 (0.6%) | 73 (0.8%) | 37 (1.2%) |
| Missing | 8 (0.1%) | 5 (0.1%) | 3 (0.1%) |

Data are presented as mean (standard deviation) or frequency (%). *Abbreviations:* please see Table S1.

**Table S12.** Factors of interest by smoking history

|  | **Never** | **Former** | **Current** |
| --- | --- | --- | --- |
|  | N=13,348 | N=9,506 | N=3,094 |
| Respiratory disease | 2,669 (20.0%) | 2,568 (27.0%) | 1,227 (39.7%) |
| Asthma | 1,016 (7.6%) | 828 (8.7%) | 181 (5.9%) |
| Chronic airflow limitation | 957 (7.2%) | 1,087 (11.4%) | 662 (21.4%) |
| Emphysema (HRCT) | 282 (2.1%) | 611 (6.4%) | 534 (17.3%) |
| Chronic bronchitis | 285 (2.1%) | 274 (2.9%) | 148 (4.8%) |
| Chronic rhinosinuitis | 266 (2.0%) | 208 (2.2%) | 90 (2.9%) |
| Restrictive spirometry pattern | 248 (1.9%) | 153 (1.6%) | 65 (2.1%) |
| Other respiratory disease | 143 (1.1%) | 137 (1.4%) | 40 (1.3%) |
| Body mass index (kg/m2) |  |  |  |
| <25.0 | 5,178 (38.8%) | 3,099 (32.6%) | 1,106 (35.7%) |
| 25-29.9 | 5,732 (42.9%) | 4,189 (44.1%) | 1,329 (43.0%) |
| ≥30 | 2,438 (18.3%) | 2,218 (23.3%) | 659 (21.3%) |
| Anemia | 440 (3.3%) | 350 (3.7%) | 96 (3.1%) |
| Peripheral arterial insufficiency | 26 (0.2%) | 29 (0.3%) | 18 (0.6%) |
| Stress level |  |  |  |
| Never stress | 652 (4.9%) | 428 (4.5%) | 205 (6.6%) |
| Any stress period | 4,768 (35.7%) | 3,390 (35.7%) | 1,103 (35.6%) |
| Some stress periods (last 5 yrs) | 5,292 (39.6%) | 3,708 (39.0%) | 1,082 (35.0%) |
| Constant stress (last 1 year) | 1,314 (9.8%) | 985 (10.4%) | 326 (10.5%) |
| Constant stress (last 5 yrs) | 1,322 (9.9%) | 995 (10.5%) | 378 (12.2%) |
| Depression | 1,581 (11.8%) | 1,482 (15.6%) | 637 (20.6%) |
| Cardiac disease | 1,022 (7.7%) | 1,012 (10.6%) | 348 (11.2%) |
| Coronary artery stenosis | 566 (4.5%) | 577 (6.5%) | 234 (8.1%) |
| Atrial fibrillation/flutter | 230 (1.7%) | 185 (1.9%) | 43 (1.4%) |
| Ischemic heart disease | 197 (1.5%) | 236 (2.5%) | 67 (2.2%) |
| Myocardial infarction | 145 (1.1%) | 194 (2.0%) | 57 (1.8%) |
| Prior CABG/PCI | 117 (0.9%) | 120 (1.3%) | 29 (0.9%) |
| Cardiac valvular disease | 101 (0.8%) | 84 (0.9%) | 22 (0.7%) |
| Heart failure | 39 (0.3%) | 67 (0.7%) | 13 (0.4%) |
| CAC total score | 0.0 (0.0-9.0) | 0.0 (0.0-28.0) | 0.0 (0.0-49.0) |
| CAC category |  |  |  |
| 0 | 8,439 (63.2%) | 5,159 (54.3%) | 1,529 (49.4%) |
| 1-100 | 3,483 (26.1%) | 2,776 (29.2%) | 1,002 (32.4%) |
| >100 | 1,188 (8.9%) | 1,311 (13.8%) | 504 (16.3%) |
| Missing | 238 (1.8%) | 260 (2.7%) | 59 (1.9%) |

**Neversmokers**

**Table S13.** Characteristics by the presence of breathlessness in neversmokers

|  | **Without breathlessness**  **(mMRC 0-1)** | **With breathlessness (mMRC ≥ 2)** |
| --- | --- | --- |
|  | N=12,987 | N=361 |
| Age (years) | 56.9 (4.3) | 57.6 (4.4) |
| Female sex | 6,171 (47.5%) | 250 (69.3%) |
| Smoking history |  |  |
| Never | 12,987 (100.0%) | 361 (100.0%) |
| Former | - | - |
| Current | - | - |
| Pack-years of smoking | 0 | 0 |
| Body mass index (kg/m^2^) |  |  |
| <25.0 | 5,132 (39.5%) | 46 (12.7%) |
| 25-29.9 | 5,631 (43.4%) | 101 (28.0%) |
| ≥30 | 2,224 (17.1%) | 214 (59.3%) |
| FEV_1_ (l) | 3.4 (0.8) | 2.8 (0.7) |
| FEV_1_ (%pred) | 103.7 (12.8) | 95.3 (15.5) |
| FVC (l) | 4.3 (1.0) | 3.5 (0.9) |
| FVC (%pred) | 103.0 (12.5) | 95.3 (13.8) |
| FEV_1_/FVC | 0.8 (0.1) | 0.8 (0.1) |
| FEV_1_/FVC (%pred) | 100.3 (6.9) | 99.5 (8.4) |
| Highest completed education |  |  |
| University | 6,962 (53.6%) | 134 (37.1%) |
| Secondary | 5,326 (41.0%) | 173 (47.9%) |
| Primary or none | 677 (5.2%) | 53 (14.7%) |
| Missing | 22 (0.2%) | 1 (0.3%) |
| Residence |  |  |
| Own house | 7,154 (55.1%) | 140 (38.8%) |
| Own apartment | 3,662 (28.2%) | 109 (30.2%) |
| Rented apartment | 2,089 (16.1%) | 108 (29.9%) |
| Other | 74 (0.6%) | 4 (1.1%) |
| Missing | 8 (0.1%) | 0 (0.0%) |

Data are presented as mean (standard deviation) or frequency (%). *Abbreviations:* please see Table S1.

**Table S14.** Underlying conditions in relation to breathlessness in never-smokers

|  | **Without breathlessness**  **(mMRC 0-1)** | **With breathlessness**  **(mMRC ≥ 2)** | **Association with breathlessness**  Odds ratio (95% CI) | |
| --- | --- | --- | --- | --- |
|  |  |  | **Crude** | **Adjusted for confounders*** |
| Respiratory disease | 2,519 (19.4%) | 150 (41.6%) | 3.0 (2.4-3.6) | 3.1 (2.6-3.8) |
| Asthma | 934 (7.2%) | 82 (22.7%) | 3.8 (3.1-4.6) | 3.5 (2.6-4.6) |
| Chronic airflow limitation | 924 (7.1%) | 33 (9.1%) | 1.3 (1.0-1.8) | 2.0 (1.6-2.5) |
| Emphysema (HRCT) | 274 (2.1%) | 8 (2.2%) | 1.1 (0.5-2.1) | 1.3 (0.7-2.3) |
| Chronic bronchitis | 256 (2.0%) | 29 (8.0%) | 4.3 (2.6- 7.2) | 3.9 (2.3- 6.7) |
| Chronic rhinosinuitis | 244 (1.9%) | 22 (6.1%) | 3.4 (2.1-5.3) | 2.8 (1.5-5.3) |
| Restrictive spirometry pattern | 226 (1.7%) | 22 (6.1%) | 3.7 (2.7-5.0) | 3.0 (2.2-4.1) |
| Other respiratory disease | 127 (1.0%) | 16 (4.4%) | 4.7 (2.6-8.5) | 4.2 (2.2-8.2) |
| Body mass index (kg/m^2^) |  |  |  |  |
| <25.0 | 5,132 (39.5%) | 46 (12.7%) | 1 (Ref) | 1 (Ref) |
| 25-29.9 | 5,631 (43.4%) | 101 (28.0%) | 2.0 (1.3-3.1) | 2.3 (1.5-3.6) |
| ≥30 | 2,224 (17.1%) | 214 (59.3%) | 10.7 (8.4-13.7) | 11.0 (8.4-14.5) |
| Anemia | 424 (3.3%) | 16 (4.4%) | 1.4 (0.8- 2.3) | 1.5 (0.9-2.5) |
| Peripheral arterial insufficiency | 26 (0.2%) | 0 (0.0%) | - | - |
| Stress level |  |  |  |  |
| Never stress | 645 (5.0%) | 7 (1.9%) | 1 (Ref) | 1 (Ref) |
| Any stress period | 4,665 (35.9%) | 103 (28.5%) | 2.0 (1.1-3.7) | 2.4 (1.3-4.6) |
| Some stress periods (last 5 yrs) | 5,193 (40.0%) | 99 (27.4%) | 1.8 (1.1-2.8) | 2.3 (1.5-3.7) |
| Constant stress (last 1 year) | 1,257 (9.7%) | 57 (15.8%) | 4.2 (2.4-7.3) | 4.8 (2.5-9.3) |
| Constant stress (last 5 yrs) | 1,227 (9.4%) | 95 (26.3%) | 7.1 (4.2-12.0) | 7.6 (4.6-12.4) |
| Depression | 1,453 (11.2%) | 128 (35.5%) | 4.4 (3.4-5.6) | 3.1 (2.5-4.0) |
| Cardiac disease | 975 (7.5%) | 47 (13.0%) | 1.8 (1.3-2.5) | 2.1 (1.5-3.0) |
| Coronary artery stenosis | 547 (4.5%) | 19 (6.3%) | 1.4 (0.9-2.1) | 1.6 (1.1-2.3) |
| Ischemic heart disease | 188 (1.4%) | 9 (2.5%) | 1.7 (0.8-3.6) | 1.9 (0.9-4.2) |
| Myocardial infarction | 138 (1.1%) | 7 (1.9%) | 1.8 (0.6-5.6) | 2.1 (0.7-6.4) |
| CABG/PCI | 111 (0.9%) | 6 (1.7%) | 2.0 (1.3-3.0) | 2.3 (1.3-4.0) |
| Atrial fibrillation/flutter | 216 (1.7%) | 14 (3.9%) | 2.4 (1.6-3.5) | 2.5 (1.6-3.9) |
| Cardiac valvular disease | 93 (0.7%) | 8 (2.2%) | 3.1 (1.4-7.2) | 4.5 (1.7-12.0) |
| Heart failure | 31 (0.2%) | 8 (2.2%) | 9.5 (3.8-23.7) | 11.4 (4.5-28.7) |
| CAC total score | 0.0 (0.0-9.0) | 0.0 (0.0-6.0) | - | - |
| CAC category |  |  |  |  |
| 0 | 8,202 (63.2%) | 237 (65.7%) | 1 (Ref) | 1 (Ref) |
| 1-100 | 3,396 (26.1%) | 87 (24.1%) | 0.9 (0.7-1.1) | 0.9 (0.7-1.1) |
| >100 | 1,159 (8.9%) | 29 (8.0%) | 0.9 (0.5-1.6) | 1.0 (0.5-1.7) |
| Missing | 230 (1.8%) | 8 (2.2%) | - | - |

Data presented as frequency (%) and associations as odds ratio (95% confidence interval) analyzed using logistic regression.

^*^Adjusted for age, sex, smoking history, pack-years of smoking, highest completed education, and body mass index (when appropriate).

*Abbreviations:* please see Table S1.

**Table S15.** Population attributable fractions of breathlessness related to underlying medical conditions in never-smokers

| **Condition** | **PAF (95% CI)** |
| --- | --- |
| Overweight and obesity | 0.690 (0.596-0.762) |
| Stress | 0.660 (0.454-0.789) |
| Respiratory disease | 0.267 (0.214-0.316) |
| Asthma | 0.151 (0.107-0.194) |
| Chronic bronchitis | 0.056 (0.022-0.088) |
| CAL | 0.044 (0.026-0.061) |
| Restrictive spirometry pattern | 0.038 (0.023-0.053) |
| Chronic rhinosinuitis | 0.036 (0.005-0.065) |
| Emphysema (on HRCT) | 0.005 (-0.007-0.017) |
| Other respiratory disease | 0.031 (0.010-0.053) |
| Depression | 0.226 (0.171-0.279) |
| Cardiac disease | 0.064 (0.024-0.103) |
| Atrial fibrillation/flutter | 0.022 (0.009-0.035) |
| Coronary artery stenosis | 0.022 (0.000-0.043) |
| Heart failure | 0.019 (0.005-0.033) |
| Cardiac valvular disease | 0.017 (-0.001-0.034) |
| Ischemic heart disease | 0.011 (-0.005-0.028) |
| Myocardial infarction | 0.009 (-0.010-0.028) |
| CABG/PCI | 0.009 (0.000-0.017) |
| CAC | -0.029 (-0.088-0.027) |
| Anemia | 0.015 (-0.005-0.034) |
| Peripheral arterial insufficiency | - |

For explanations and abbreviations, see Table 3 in the main article.

*Adjusted for age, sex, smoking history, pack-years, highest education and BMI (except when BMI is analyzed as factor).

**Former smokers**

**Table S16.** Characteristics by the presence of breathlessness in former smokers

|  | **Without breathlessness**  **(mMRC 0-1)** | **With breathlessness (mMRC ≥ 2)** |
| --- | --- | --- |
|  | N=9,092 | N=414 |
| Age (years) | 58.3 (4.3) | 59.1 (4.3) |
| Female sex | 4,965 (54.6%) | 293 (70.8%) |
| Smoking history |  |  |
| Never | - | - |
| Former | 9,092 (100.0%) | 414 (100.0%) |
| Current | - | - |
| Pack-years of smoking | 12.7 (11.1) | 19.5 (15.4) |
| Body mass index (kg/m^2^) |  |  |
| <25.0 | 3,051 (33.6%) | 48 (11.6%) |
| 25-29.9 | 4,066 (44.7%) | 123 (29.7%) |
| ≥30 | 1,975 (21.7%) | 243 (58.7%) |
| FEV_1_ (l) | 3.2 (0.7) | 2.7 (0.7) |
| FEV_1_ (%pred) | 103.0 (13.5) | 92.8 (16.3) |
| FVC (l) | 4.2 (0.9) | 3.6 (0.8) |
| FVC (%pred) | 103.8 (12.7) | 96.9 (13.5) |
| FEV_1_/FVC | 0.8 (0.1) | 0.8 (0.1) |
| FEV_1_/FVC (%pred) | 98.9 (7.7) | 95.4 (11.6) |
| Highest completed education |  |  |
| University | 3,822 (42.0%) | 111 (26.8%) |
| Secondary | 4,328 (47.6%) | 222 (53.6%) |
| Primary or none | 922 (10.1%) | 80 (19.3%) |
| Missing | 20 (0.2%) | 1 (0.2%) |
| Residence |  |  |
| Own house | 4,001 (44.0%) | 155 (37.4%) |
| Own apartment | 2,909 (32.0%) | 122 (29.5%) |
| Rented apartment | 2,109 (23.2%) | 132 (31.9%) |
| Other | 68 (0.7%) | 5 (1.2%) |
| Missing | 5 (0.1%) | 0 (0.0%) |

Data are presented as mean (standard deviation) or frequency (%). *Abbreviations:* please see Table S1.

**Table S17.** Underlying conditions in relation to breathlessness in former smokers

|  | **Without breathlessness**  **(mMRC 0-1)** | **With breathlessness**  **(mMRC ≥ 2)** | **Association with breathlessness**  Odds ratio (95% CI) | |
| --- | --- | --- | --- | --- |
|  |  |  | **Crude** | **Adjusted for confounders*** |
| Respiratory disease | 2,362 (26.0%) | 206 (49.8%) | 2.8 (2.0-4.0) | 2.4 (1.7-3.6) |
| Asthma | 743 (8.2%) | 85 (20.5%) | 2.9 (2.5-3.3) | 2.6 (2.2-3.0) |
| Chronic airflow limitation | 999 (11.0%) | 88 (21.3%) | 2.2 (2.0-2.5) | 2.0 (1.7-2.4) |
| Emphysema (HRCT) | 556 (6.1%) | 55 (13.3%) | 2.4 (1.2-4.4) | 1.9 (0.8-4.2) |
| Chronic bronchitis | 228 (2.5%) | 46 (11.1%) | 4.9 (3.8-6.2) | 4.6 (3.7-5.7) |
| Chronic rhinosinuitis | 188 (2.1%) | 20 (4.8%) | 2.4 (1.4-4.3) | 2.0 (1.0-4.2) |
| Restrictive spirometry pattern | 132 (1.5%) | 21 (5.1%) | 3.6 (2.1-6.4) | 2.8 (1.7-4.5) |
| Other respiratory disease | 120 (1.3%) | 17 (4.1%) | 3.2 (1.6-6.3) | 3.3 (1.7-6.4) |
| Body mass index (kg/m^2^) |  |  |  |  |
| <25.0 | 3,051 (33.6%) | 48 (11.6%) | 1 (Ref) | 1 (Ref) |
| 25-29.9 | 4,066 (44.7%) | 123 (29.7%) | 1.9 (1.3-2.8) | 2.0 (1.4-2.9) |
| ≥30 | 1,975 (21.7%) | 243 (58.7%) | 7.8 (6.5-9.4) | 7.2 (6.0-8.6) |
| Anemia | 327 (3.6%) | 23 (5.6%) | 1.6 (1.3-2.0) | 1.7 (1.2-2.4) |
| Peripheral arterial insufficiency | 25 (0.3%) | 4 (1.0%) | 3.5 (0.9-13.2) | 3.4 (0.9- 12.6) |
| Stress level |  |  |  |  |
| Never stress | 418 (4.6%) | 10 (2.4%) | 1 (Ref) | 1 (Ref) |
| Any stress period | 3,287 (36.2%) | 103 (24.9%) | 1.3 (0.6-2.7) | 1.6 (0.7-3.4) |
| Some stress periods (last 5 yrs) | 3,566 (39.2%) | 142 (34.3%) | 1.7 (0.8-3.4) | 2.3 (1.0-5.3) |
| Constant stress (last 1 year) | 931 (10.2%) | 54 (13.0%) | 2.4 (1.3-4.6) | 3.1 (1.5-6.4) |
| Constant stress (last 5 yrs) | 890 (9.8%) | 105 (25.4%) | 4.9 (2.5-9.8) | 6.1 (2.6-14.0) |
| Depression | 1,340 (14.7%) | 142 (34.3%) | 3.0 (2.4-3.7) | 2.5 (2.1-2.9) |
| Cardiac disease | 916 (10.1%) | 96 (23.2%) | 2.7 (2.0-3.7) | 2.6 (1.9-3.7) |
| Coronary artery stenosis | 538 (6.4%) | 39 (10.8%) | 1.8 (1.5-2.2) | 1.7 (1.5-1.9) |
| Ischemic heart disease | 203 (2.2%) | 33 (8.0%) | 3.8 (2.6-5.6) | 2.7 (1.8-4.1) |
| Myocardial infarction | 166 (1.8%) | 28 (6.8%) | 3.9 (2.4-6.3) | 2.7 (1.6-4.3) |
| CABG/PCI | 107 (1.2%) | 13 (3.1%) | 2.7 (1.4-5.2) | 1.9 (0.9-4.0) |
| Atrial fibrillation/flutter | 165 (1.8%) | 20 (4.8%) | 2.7 (1.4-5.4) | 2.7 (1.3-5.6) |
| Cardiac valvular disease | 72 (0.8%) | 12 (2.9%) | 3.7 (1.7-8.4) | 4.1 (2.0-8.6) |
| Heart failure | 51 (0.6%) | 16 (3.9%) | 7.1 (4.0-12.8) | 5.5 (3.2-9.5) |
| CAC total score | 0.0 (0.0-27.0) | 1.0 (0.0-72.0) | - | - |
| CAC category |  |  |  |  |
| 0 | 4,971 (54.7%) | 188 (45.4%) | 1 (Ref) | 1 (Ref) |
| 1-100 | 2,661 (29.3%) | 115 (27.8%) | 1.1 (0.8-1.5) | 1.1 (0.7-1.5) |
| >100 | 1,228 (13.5%) | 83 (20.0%) | 1.8 (1.4-2.3) | 1.6 (1.3-1.9) |
| Missing | 232 (2.6%) | 28 (6.8%) | - | - |

Data presented as frequency (%) and associations as odds ratio (95% confidence interval) analyzed using logistic regression.

^*^Adjusted for age, sex, smoking history, pack-years of smoking, highest completed education, and body mass index (when appropriate).

*Abbreviations:* please see Table S1.

**Table S18.** Population attributable fractions of breathlessness related to underlying medical conditions in former smokers

| **Condition** | **PAF (95% CI)** |
| --- | --- |
| Overweight and obesity | 0.637 (0.552-0.706) |
| Stress | 0.550 (0.078-0.780) |
| Respiratory disease | 0.269 (0.135-0.383) |
| Asthma | 0.113 (0.087-0.138) |
| CAL | 0.095 (0.067-0.122) |
| Emphysema (on HRCT) | 0.055 (-0.029-0.133) |
| Chronic bronchitis | 0.078 (0.063-0.093) |
| Chronic rhinosinuitis | 0.021 (-0.007-0.049) |
| Restrictive spirometry pattern | 0.029 (0.011-0.047) |
| Other respiratory disease | 0.025 (0.006-0.044) |
| Depression | 0.185 (0.146-0.222) |
| Cardiac disease | 0.130 (0.073-0.184) |
| Ischemic heart disease | 0.043 (0.019-0.068) |
| Myocardial infarction | 0.036 (0.012-0.059) |
| CABG/PCI | 0.013 (-0.006-0.031) |
| Coronary artery stenosis | 0.039 (0.026-0.053) |
| Heart failure | 0.028 (0.015-0.041) |
| Atrial fibrillation/flutter | 0.027 (-0.001-0.054) |
| Cardiac valvular disease | 0.020 (0.005-0.035) |
| CAC | 0.084 (-0.022-0.179) |
| Anemia | 0.020 (0.004-0.035) |
| Peripheral arterial insufficiency | 0.006 (-0.004-0.017) |

For explanations and abbreviations, see Table 3 in the main article.

*Adjusted for age, sex, smoking history, pack-years, highest education and BMI (except when BMI is analyzed as factor).

**Current smokers**

**Table S19.** Characteristics by the presence of breathlessness in current smokers

|  | **Without breathlessness**  **(mMRC 0-1)** | **With breathlessness (mMRC ≥ 2)** |
| --- | --- | --- |
|  | N=2,917 | N=177 |
| Age (years) | 57.3 (4.2) | 58.1 (4.3) |
| Female sex | 1,483 (50.8%) | 103 (58.2%) |
| Smoking history |  |  |
| Never | - | - |
| Former | - | - |
| Current | 2,917 (100.0%) | 177 (100.0%) |
| Pack-years of smoking | 21.8 (14.1) | 30.6 (17.2) |
| Body mass index (kg/m^2^) |  |  |
| <25.0 | 1,072 (36.8%) | 34 (19.2%) |
| 25-29.9 | 1,260 (43.2%) | 69 (39.0%) |
| ≥30 | 585 (20.1%) | 74 (41.8%) |
| FEV_1_ (l) | 3.1 (0.8) | 2.6 (0.8) |
| FEV_1_ (%pred) | 98.7 (14.8) | 86.3 (19.8) |
| FVC (l) | 4.2 (1.0) | 3.7 (1.0) |
| FVC (%pred) | 102.5 (13.2) | 95.7 (14.9) |
| FEV_1_/FVC | 0.8 (0.1) | 0.7 (0.1) |
| FEV_1_/FVC (%pred) | 96.0 (9.4) | 89.4 (14.1) |
| Highest completed education |  |  |
| University | 908 (31.1%) | 31 (17.5%) |
| Secondary | 1,545 (53.0%) | 99 (55.9%) |
| Primary or none | 452 (15.5%) | 47 (26.6%) |
| Missing | 12 (0.4%) | 0 (0.0%) |
| Residence |  |  |
| Own house | 950 (32.6%) | 34 (19.2%) |
| Own apartment | 876 (30.0%) | 46 (26.0%) |
| Rented apartment | 1,054 (36.1%) | 94 (53.1%) |
| Other | 34 (1.2%) | 3 (1.7%) |
| Missing | 3 (0.1%) | 0 (0.0%) |

Data are presented as mean (standard deviation) or frequency (%). *Abbreviations:* please see Table S1.

**Table S20.** Underlying conditions in relation to breathlessness in current smokers

|  | **Without breathlessness**  **(mMRC 0-1)** | **With breathlessness**  **(mMRC ≥ 2)** | **Association with breathlessness**  Odds ratio (95% CI) | |
| --- | --- | --- | --- | --- |
|  |  |  | **Crude** | **Adjusted for confounders*** |
| Respiratory disease | 1,113 (38.2%) | 114 (64.4%) | 2.9 (2.4-3.5) | 2.7 (2.1-3.4) |
| Asthma | 159 (5.5%) | 22 (12.4%) | 2.5 (1.8-3.3) | 2.4 (1.8-3.2) |
| Chronic airflow limitation | 584 (20.0%) | 78 (44.1%) | 3.1 (2.2-4.5) | 2.9 (2.0-4.4) |
| Emphysema (HRCT) | 484 (16.6%) | 50 (28.2%) | 2.0 (1.4-2.7) | 1.9 (1.3-2.8) |
| Chronic bronchitis | 120 (4.1%) | 28 (15.8%) | 4.4 (3.8-5.1) | 3.5 (2.9-4.2) |
| Chronic rhinosinuitis | 81 (2.8%) | 9 (5.1%) | 1.9 (0.9-3.9) | 1.9 (0.8-4.2) |
| Restrictive spirometry pattern | 57 (2.0%) | 8 (4.5%) | 2.4 (1.3-4.4) | 1.8 (0.8-4.1) |
| Other respiratory disease | 30 (1.0%) | 10 (5.6%) | 5.8 (2.8-11.8) | 6.7 (2.8-16.2) |
| Body mass index (kg/m^2^) |  |  |  |  |
| <25.0 | 1,072 (36.8%) | 34 (19.2%) | 1 (Ref) | 1 (Ref) |
| 25-29.9 | 1,260 (43.2%) | 69 (39.0%) | 1.7 (1.3-2.2) | 1.9 (1.4-2.4) |
| ≥30 | 585 (20.1%) | 74 (41.8%) | 4.0 (3.1-5.2) | 3.9 (2.8-5.4) |
| Anemia | 86 (2.9%) | 10 (5.6%) | 2.0 (0.8-5.0) | 2.4 (0.9-6.5) |
| Peripheral arterial insufficiency | 14 (0.5%) | 4 (2.3%) | 4.8 (1.5-15.1) | 4.1 (1.3-12.8) |
| Stress level |  |  |  |  |
| Never stress | 200 (6.9%) | 5 (2.8%) | 1 (Ref) | 1 (Ref) |
| Any stress period | 1,054 (36.1%) | 49 (27.7%) | 1.9 (0.7-5.3) | 2.1 (0.7-6.3) |
| Some stress periods (last 5 yrs) | 1,040 (35.7%) | 42 (23.7%) | 1.6 (0.5-5.1) | 1.9 (0.5-6.6) |
| Constant stress (last 1 year) | 291 (10.0%) | 35 (19.8%) | 4.8 (1.6-15.6) | 5.8 (1.7- 19.3) |
| Constant stress (last 5 yrs) | 332 (11.4%) | 46 (26.0%) | 5.5 (2.1-14.6) | 6.5 (2.4-17.6) |
| Depression | 557 (19.1%) | 80 (45.2%) | 3.5 (2.4-5.12) | 3.3 (2.1-5.2) |
| Cardiac disease | 313 (10.7%) | 35 (19.8%) | 2.1 (1.3-3.1) | 1.9 (1.2-2.8) |
| Coronary artery stenosis | 216 (7.9%) | 18 (11.8%) | 1.5 (0.9-2.8) | 1.4 (0.9-2.3) |
| Ischemic heart disease | 56 (1.9%) | 11 (6.2%) | 3.4 (1.9-5.9) | 2.1 (1.1-4.3 |
| Myocardial infarction | 47 (1.6%) | 10 (5.6%) | 3.7 (2.1-6.4) | 2.2 (1.1- 4.3) |
| CABG/PCI | 24 (0.8%) | 5 (2.8%) | 3.5 (1.0-12.2) | 2.6 (0.6-10.5) |
| Atrial fibrillation/flutter | 36 (1.2%) | 7 (4.0%) | 3.3 (2.2-4.9) | 2.9 (1.7-4.7) |
| Cardiac valvular disease | 19 (0.7%) | 3 (1.7%) | 2.6 (0.6-11.5) | 2.9 (0.5-15.6) |
| Heart failure | 11 (0.4%) | 2 (1.1%) | 3.0 (0.4-22.0) | 2.4 (0.3-19.1) |
| CAC total score | 0.0 (0.0-46.0) | 8.0 (0.0-84.0) | - | - |
| CAC category |  |  |  |  |
| 0 | 1,465 (50.2%) | 64 (36.2%) | 1 (Ref) | 1 (Ref) |
| 1-100 | 937 (32.1%) | 65 (36.7%) | 1.6 (1.1-2.4) | 1.4 (0.8-2.1) |
| >100 | 464 (15.9%) | 40 (22.6%) | 2.0 (1.3-3.1) | 1.5 (0.9-2.5) |
| Missing | 51 (1.7%) | 8 (4.5%) | - | - |

Data presented as frequency (%) and associations as odds ratio (95% confidence interval) analyzed using logistic regression.

^*^Adjusted for age, sex, smoking history, pack-years of smoking, highest completed education, and body mass index (when appropriate).

*Abbreviations:* please see Table S1.

**Table S21.** Population attributable fractions of breathlessness related to underlying medical conditions in current smokers

| **Condition** | **PAF (95% CI)** |
| --- | --- |
| Stress | 0.610 (-0.093-0.861) |
| Overweight and obesity | 0.468 (0.374-0.547) |
| Respiratory disease | 0.375 (0.286-0.453) |
| Asthma | 0.065 (0.038-0.090) |
| CAL | 0.268 (0.140-0.377) |
| Emphysema (on HRCT) | 0.124 (0.041-0.199) |
| Chronic bronchitis | 0.100 (0.083-0.117) |
| Chronic rhinosinuitis | 0.022 (-0.014-0.056) |
| Restrictive spirometry pattern | 0.017 (-0.014-0.048) |
| Other respiratory disease | 0.043 (0.012-0.073) |
| Depression | 0.290 (0.153-0.404) |
| Anemia | 0.031 (-0.017-0.077) |
| CAC | 0.160 (-0.060-0.334) |
| Cardiac disease | 0.081 (0.015-0.143) |
| Coronary artery stenosis | 0.032 (-0.017-0.080) |
| Ischemic heart disease | 0.028 (-0.004-0.059) |
| Myocardial infarction | 0.026 (-0.001-0.052) |
| CABG/PCI | 0.015 (-0.015-0.044) |
| Atrial fibrillation/flutter | 0.023 (0.009-0.037) |
| Cardiac valvular disease | 0.010 (-0.013-0.033) |
| Heart failure | 0.006 (-0.013-0.024) |
| Peripheral arterial insufficiency | 0.016 (-0.002-0.033) |

For explanations and abbreviations, see Table 3 in the main article.

*Adjusted for age, sex, smoking history, pack-years, highest education and BMI (except when BMI is analyzed as factor).

**Subgroup analysis by: SELF-REPORTED CARDIORESPIRATORY DISEASE**

**Table S22.** Characteristics by presence of self-reported cardiorespiratory disease

|  | **Without self-reported cardiorespiratory disease** | **With self-reported cardiorespiratory disease** |
| --- | --- | --- |
|  | N=21,572 | N=4,376 |
| Age (years) | 57.4 (4.3) | 58.0 (4.4) |
| Female sex | 11,113 (51.5%) | 2,152 (49.2%) |
| Smoking history |  |  |
| Never | 11,352 (52.6%) | 1,996 (45.6%) |
| Former | 7,693 (35.7%) | 1,813 (41.4%) |
| Current | 2,527 (11.7%) | 567 (13.0%) |
| Pack-years of smoking | 6.9 (11.1) | 9.8 (14.3) |
| Body mass index (kg/m2) |  |  |
| <25.0 | 8,046 (37.3%) | 1,337 (30.6%) |
| 25-29.9 | 9,357 (43.4%) | 1,893 (43.3%) |
| ≥30 | 4,169 (19.3%) | 1,146 (26.2%) |
| FEV_1_ (l) | 3.3 (0.8) | 3.1 (0.8) |
| FEV_1_ (%pred) | 103.5 (13.1) | 97.5 (15.4) |
| FVC (l) | 4.2 (1.0) | 4.1 (1.0) |
| FVC (%pred) | 103.5 (12.6) | 100.8 (13.6) |
| FEV_1_/FVC | 0.8 (0.1) | 0.8 (0.1) |
| FEV_1_/FVC (%pred) | 99.7 (7.2) | 96.4 (9.9) |
| Highest completed education |  |  |
| University | 10,026 (46.5%) | 1,942 (44.4%) |
| Secondary | 9,699 (45.0%) | 1,994 (45.6%) |
| Primary or none | 1,803 (8.4%) | 428 (9.8%) |
| Missing | 44 (0.2%) | 12 (0.3%) |
| Residence |  |  |
| Own house | 10,476 (48.6%) | 1,958 (44.7%) |
| Own apartment | 6,471 (30.0%) | 1,253 (28.6%) |
| Rented apartment | 4,470 (20.7%) | 1,116 (25.5%) |
| Other | 140 (0.6%) | 48 (1.1%) |
| Missing | 15 (0.1%) | 1 (0.0%) |

**Table S23.** Factors of interest by presence of self-reported cardiorespiratory disease

|  | **Without self-reported cardiorespiratory disease** | **With self-reported cardiorespiratory disease** |
| --- | --- | --- |
|  | N=21,572 | N=4,376 |
| Respiratory disease | 2,894 (13.4%) | 3,570 (81.6%) |
| Asthma | 0 (0.0%) | 2,025 (46.3%) |
| Chronic airflow limitation | 1,824 (8.5%) | 882 (20.2%) |
| Emphysema (HRCT) | 1,070 (5.0%) | 357 (8.2%) |
| Chronic bronchitis | 0 (0.0%) | 707 (16.2%) |
| Chronic rhinosinuitis | 0 (0.0%) | 564 (12.9%) |
| Restrictive spirometry pattern | 327 (1.5%) | 139 (3.2%) |
| Other respiratory disease | 0 (0.0%) | 320 (7.3%) |
| Body mass index (kg/m2) |  |  |
| <25.0 | 8,046 (37.3%) | 1,337 (30.6%) |
| 25-29.9 | 9,357 (43.4%) | 1,893 (43.3%) |
| ≥30 | 4,169 (19.3%) | 1,146 (26.2%) |
| Anemia | 708 (3.3%) | 178 (4.1%) |
| Peripheral arterial insufficiency | 44 (0.2%) | 29 (0.7%) |
| Stress level |  |  |
| Never stress | 1,105 (5.1%) | 180 (4.1%) |
| Any stress period | 7,905 (36.6%) | 1,356 (31.0%) |
| Some stress periods (last 5 yrs) | 8,398 (38.9%) | 1,684 (38.5%) |
| Constant stress (last 1 year) | 2,109 (9.8%) | 516 (11.8%) |
| Constant stress (last 5 yrs) | 2,055 (9.5%) | 640 (14.6%) |
| Depression | 2,874 (13.3%) | 826 (18.9%) |
| Cardiac disease | 1,062 (4.9%) | 1,320 (30.2%) |
| Coronary artery stenosis | 1,062 (5.2%) | 315 (8.0%) |
| Atrial fibrillation/flutter | 0 (0.0%) | 458 (10.5%) |
| Ischemic heart disease | 0 (0.0%) | 500 (11.4%) |
| Myocardial infarction | 0 (0.0%) | 396 (9.0%) |
| Prior CABG/PCI | 0 (0.0%) | 266 (6.1%) |
| Heart failure | 0 (0.0%) | 119 (2.7%) |
| Cardiac valvular disease | 0 (0.0%) | 207 (4.7%) |
| CAC total score | 0.0 (0.0-18.0) | 0.0 (0.0-30.0) |
| CAC category |  |  |
| 0 | 12,890 (59.8%) | 2,237 (51.1%) |
| 1-100 | 6,133 (28.4%) | 1,128 (25.8%) |
| >100 | 2,384 (11.1%) | 619 (14.1%) |
| Missing | 165 (0.8%) | 392 (9.0%) |

*Abbreviations:* please see Table S1.

**Without self-reported cardiorespiratory disease**

**Table S24.** Characteristics by the presence of breathlessness in people without self-reported cardiorespiratory disease

|  | **Without breathlessness**  **(mMRC 0-1)** | **With breathlessness (mMRC ≥ 2)** |
| --- | --- | --- |
|  | N=21,063 | N=509 |
| Age (years) | 57.4 (4.3) | 58.1 (4.4) |
| Female sex | 10,763 (51.1%) | 350 (68.8%) |
| Smoking history |  |  |
| Never | 11,137 (52.9%) | 215 (42.2%) |
| Former | 7,482 (35.5%) | 211 (41.5%) |
| Current | 2,444 (11.6%) | 83 (16.3%) |
| Pack-years of smoking | 6.8 (11.0) | 11.8 (14.8) |
| Body mass index (kg/m^2^) |  |  |
| <25.0 | 7,991 (37.9%) | 55 (10.8%) |
| 25-29.9 | 9,212 (43.7%) | 145 (28.5%) |
| ≥30 | 3,860 (18.3%) | 309 (60.7%) |
| FEV_1_ (l) | 3.3 (0.8) | 2.8 (0.6) |
| FEV_1_ (%pred) | 103.6 (13.0) | 96.8 (14.0) |
| FVC (l) | 4.3 (1.0) | 3.6 (0.8) |
| FVC (%pred) | 103.6 (12.6) | 97.5 (12.5) |
| FEV_1_/FVC | 0.8 (0.1) | 0.8 (0.1) |
| FEV_1_/FVC (%pred) | 99.7 (7.2) | 98.8 (8.7) |
| Highest completed education |  |  |
| University | 9,881 (46.9%) | 145 (28.5%) |
| Secondary | 9,431 (44.8%) | 268 (52.7%) |
| Primary or none | 1,709 (8.1%) | 94 (18.5%) |
| Missing | 42 (0.2%) | 2 (0.4%) |
| Residence |  |  |
| Own house | 10,298 (48.9%) | 178 (35.0%) |
| Own apartment | 6,316 (30.0%) | 155 (30.5%) |
| Rented apartment | 4,299 (20.4%) | 171 (33.6%) |
| Other | 135 (0.6%) | 5 (1.0%) |
| Missing | 15 (0.1%) | 0 (0.0%) |

Data are presented as mean (standard deviation) or frequency (%). *Abbreviations:* please see Table S1.

**Table S25.** Underlying conditions in relation to breathlessness in people without self-reported cardiorespiratory disease

|  | **Without breathlessness**  **(mMRC 0-1)** | **With breathlessness**  **(mMRC ≥ 2)** | **Association with breathlessness**  Odds ratio (95% CI) | |
| --- | --- | --- | --- | --- |
|  |  |  | **Crude** | **Adjusted for confounders*** |
| Respiratory disease | 2,804 (13.3%) | 90 (17.7%) | 1.4 (1.1-1.8) | 1.3 (0.9-2.0) |
| Asthma | 0 (0.0%) | 0 (0.0%) | - | - |
| Chronic airflow limitation | 1,773 (8.4%) | 51 (10.0%) | 1.2 (0.9-1.7) | 1.2 (0.8-1.9) |
| Emphysema (HRCT) | 1,035 (4.9%) | 35 (6.9%) | 1.4 (0.9-2.4) | 1.2 (0.7-2.2) |
| Chronic bronchitis | 0 (0.0%) | 0 (0.0%) | - | - |
| Chronic rhinosinuitis | 0 (0.0%) | 0 (0.0%) | - | - |
| Restrictive spirometry pattern | 307 (1.5%) | 20 (3.9%) | 2.8 (1.8-4.2) | 2.0 (1.3-3.2) |
| Other respiratory disease | 0 (0.0%) | 0 (0.0%) | - | - |
| Body mass index (kg/m^2^) |  |  |  |  |
| <25.0 | 7,991 (37.9%) | 55 (10.8%) | 1 (Ref) | 1 (Ref) |
| 25-29.9 | 9,212 (43.7%) | 145 (28.5%) | 2.3 (2.0-2.6) | 2.5 (2.1-2.9) |
| ≥30 | 3,860 (18.3%) | 309 (60.7%) | 11.6 (10.3-13.1) | 11.2 (9.9-12.7) |
| Anemia | 685 (3.3%) | 23 (4.5%) | 1.4 (0.9-2.3) | 1.6 (1.1-2.5) |
| Peripheral arterial insufficiency | 41 (0.2%) | 3 (0.6%) | 3.0 (1.2-7.5) | 3.0 (1.2-7.8) |
| Stress level |  |  |  |  |
| Never stress | 1,093 (5.2%) | 12 (2.4%) | 1 (Ref) | 1 (Ref) |
| Any stress period | 7,777 (36.9%) | 128 (25.1%) | 1.5 (0.8-2.7) | 1.8 (1.0-3.1) |
| Some stress periods (last 5 yrs) | 8,247 (39.2%) | 151 (29.7%) | 1.7 (1.1-2.4) | 2.2 (1.5-3.3) |
| Constant stress (last 1 year) | 2,030 (9.6%) | 79 (15.5%) | 3.5 (2.7-4.6) | 4.3 (3.2-5.7) |
| Constant stress (last 5 yrs) | 1,916 (9.1%) | 139 (27.3%) | 6.6 (5.2-8.6) | 7.3 (5.5-9.6) |
| Depression | 2,680 (12.7%) | 194 (38.1%) | 4.2 (3.2-5.6) | 3.2 (2.4-4.3) |
| Cardiac disease | 1,034 (4.9%) | 28 (5.5%) | 1.1 (0.8-1.7) | 1.1 (0.8-1.6) |
| Coronary artery stenosis | 1,034 (4.9%) | 28 (5.5%) | 1.2 (0.8-1.8) | 1.2 (0.9-1.7) |
| Ischemic heart disease | 0 (0.0%) | 0 (0.0%) | - | - |
| Myocardial infarction | 0 (0.0%) | 0 (0.0%) | - | - |
| CABG/PCI | 0 (0.0%) | 0 (0.0%) | - | - |
| Atrial fibrillation/flutter | 0 (0.0%) | 0 (0.0%) | - | - |
| Cardiac valvular disease | 0 (0.0%) | 0 (0.0%) | - | - |
| Heart failure | 0 (0.0%) | 0 (0.0%) | - | - |
| CAC total score | 0.0 (0.0-17.0) | 0.0 (0.0-23.0) | - | - |
| CAC category |  |  |  |  |
| 0 | 12,602 (59.8%) | 288 (56.6%) | 1 (Ref) | 1 (Ref) |
| 1-100 | 5,977 (28.4%) | 156 (30.6%) | 1.1 (0.9-1.4) | 1.0 (0.8-1.3) |
| >100 | 2,325 (11.0%) | 59 (11.6%) | 1.1 (0.8-1.6) | 0.9 (0.6-1.4) |
| Missing | 159 (0.8%) | 6 (1.2%) | - | - |

Data presented as frequency (%) and associations as odds ratio (95% confidence interval) analyzed using logistic regression.

^*^Adjusted for age, sex, smoking history, pack-years of smoking, highest completed education, and body mass index (when appropriate).

*Abbreviations:* please see Table S1.

**Table S26.** Population attributable fractions of breathlessness related to underlying medical conditions in people without self-reported cardiorespiratory disease

| **Condition** | **PAF (95% CI)** |
| --- | --- |
| Overweight and obesity | 0.716 (0.684-0.746) |
| Stress | 0.608 (0.447-0.722) |
| Depression | 0.247 (0.170-0.318) |
| Respiratory disease | 0.041 (-0.018-0.097) |
| Restrictive spirometry pattern | 0.019 (0.003-0.034) |
| CAL | 0.018 (-0.021-0.056) |
| Emphysema (on HRCT) | 0.012 (-0.023-0.046) |
| Asthma | - |
| Chronic bronchitis | - |
| Chronic rhinosinuitis | - |
| Other respiratory disease | - |
| Anemia | 0.016 (-0.001-0.032) |
| Peripheral arterial insufficiency | 0.004 (-0.001-0.009) |
| Cardiac disease | - |
| Coronary artery stenosis | 0.010 (-0.010-0.029) |
| Atrial fibrillation/flutter | - |
| Ischemic heart disease | - |
| Myocardial infarction | - |
| CABG/PCI | - |
| Cardiac valvular disease | - |
| Heart failure | - |
| CAC | -0.006 (-0.113-0.092) |

For explanations and abbreviations, see Table 3 in the main article.

*Adjusted for age, sex, smoking history, pack-years, highest education and BMI (except when BMI is analyzed as factor).

**With self-reported cardiorespiratory disease**

**Table S27.** Characteristics by the presence of breathlessness in people with self-reported cardiorespiratory disease

|  | **Without breathlessness**  **(mMRC 0-1)** | **With breathlessness (mMRC ≥ 2)** |
| --- | --- | --- |
|  | N=3,933 | N=443 |
| Age (years) | 58.0 (4.4) | 58.6 (4.4) |
| Female sex | 1,856 (47.2%) | 296 (66.8%) |
| Smoking history |  |  |
| Never | 1,850 (47.0%) | 146 (33.0%) |
| Former | 1,610 (40.9%) | 203 (45.8%) |
| Current | 473 (12.0%) | 94 (21.2%) |
| Pack-years of smoking | 9.0 (13.4) | 16.9 (19.3) |
| Body mass index (kg/m^2^) |  |  |
| <25.0 | 1,264 (32.1%) | 73 (16.5%) |
| 25-29.9 | 1,745 (44.4%) | 148 (33.4%) |
| ≥30 | 924 (23.5%) | 222 (50.1%) |
| FEV_1_ (l) | 3.2 (0.8) | 2.6 (0.7) |
| FEV_1_ (%pred) | 98.6 (14.6) | 87.7 (18.8) |
| FVC (l) | 4.2 (1.0) | 3.5 (0.9) |
| FVC (%pred) | 101.5 (13.2) | 94.4 (15.3) |
| FEV_1_/FVC | 0.8 (0.1) | 0.7 (0.1) |
| FEV_1_/FVC (%pred) | 96.9 (9.3) | 92.3 (13.4) |
| Highest completed education |  |  |
| University | 1,811 (46.0%) | 131 (29.6%) |
| Secondary | 1,768 (45.0%) | 226 (51.0%) |
| Primary or none | 342 (8.7%) | 86 (19.4%) |
| Missing | 12 (0.3%) | 0 (0.0%) |
| Residence |  |  |
| Own house | 1,807 (45.9%) | 151 (34.1%) |
| Own apartment | 1,131 (28.8%) | 122 (27.5%) |
| Rented apartment | 953 (24.2%) | 163 (36.8%) |
| Other | 41 (1.0%) | 7 (1.6%) |
| Missing | 1 (0.0%) | 0 (0.0%) |

Data are presented as mean (standard deviation) or frequency (%). *Abbreviations:* please see Table S1.

**Table S28.** Underlying conditions in relation to breathlessness in people with self-reported cardiorespiratory disease

|  | **Without breathlessness**  **(mMRC 0-1)** | **With breathlessness**  **(mMRC ≥ 2)** | **Association with breathlessness**  Odds ratio (95% CI) | |
| --- | --- | --- | --- | --- |
|  |  |  | **Crude** | **Adjusted for confounders*** |
| Respiratory disease | 3,190 (81.1%) | 380 (85.8%) | 1.4 (1.0-1.9) | 1.3 (0.9-1.7) |
| Asthma | 1,836 (46.7%) | 189 (42.7%) | 0.8 (0.7-1.0) | 0.9 (0.8-1.0) |
| Chronic airflow limitation | 734 (18.7%) | 148 (33.4%) | 2.2 (1.9-2.5) | 2.0 (1.8-2.4) |
| Emphysema (HRCT) | 279 (7.1%) | 78 (17.6%) | 2.8 (1.9-4.1) | 2.3 (1.3-4.1) |
| Chronic bronchitis | 604 (15.4%) | 103 (23.3%) | 1.7 (1.4-2.0) | 1.5 (1.3-1.9) |
| Chronic rhinosinuitis | 513 (13.0%) | 51 (11.5%) | 0.9 (0.6-1.3) | 0.8 (0.5-1.4) |
| Restrictive spirometry pattern | 108 (2.7%) | 31 (7.0%) | 2.7 (2.0-3.5) | 2.7 (2.0-3.6) |
| Other respiratory disease | 277 (7.0%) | 43 (9.7%) | 1.4 (0.9-2.1) | 1.6 (1.0-2.4) |
| Body mass index (kg/m^2^) |  |  |  |  |
| <25.0 | 1,264 (32.1%) | 73 (16.5%) | 1 (Ref) | 1 (Ref) |
| 25-29.9 | 1,745 (44.4%) | 148 (33.4%) | 1.5 (1.3-1.7) | 1.6 (1.4-1.8) |
| ≥30 | 924 (23.5%) | 222 (50.1%) | 4.2 (3.5-5.0) | 4.1 (3.1-5.5) |
| Anemia | 152 (3.9%) | 26 (5.9%) | 1.6 (0.9-2.5) | 1.7 (1.1-2.7) |
| Peripheral arterial insufficiency | 24 (0.6%) | 5 (1.1%) | 1.9 (0.9-3.8) | 1.8 (0.9-3.6) |
| Stress level |  |  |  |  |
| Never stress | 170 (4.3%) | 10 (2.3%) | 1 (Ref) | 1 (Ref) |
| Any stress period | 1,229 (31.2%) | 127 (28.7%) | 1.8 (0.6-5.0) | 2.1 (0.7-6.7) |
| Some stress periods (last 5 yrs) | 1,552 (39.5%) | 132 (29.8%) | 1.4 (0.5-3.8) | 2.0 (0.7-6.0) |
| Constant stress (last 1 year) | 449 (11.4%) | 67 (15.1%) | 2.5 (0.9-7.0) | 3.1 (1.0-9.9) |
| Constant stress (last 5 yrs) | 533 (13.6%) | 107 (24.2%) | 3.4 (1.2-9.9) | 4.3 (1.3-15.0) |
| Depression | 670 (17.0%) | 156 (35.2%) | 2.6 (2.3-3.0) | 2.1 (1.7-2.5) |
| Cardiac disease | 1,170 (29.7%) | 150 (33.9%) | 1.2 (0.9-1.6) | 1.3 (1.0-1.8) |
| Ischemic heart disease | 447 (11.4%) | 53 (12.0%) | 1.1 (0.9-1.3) | 0.9- (0.7-1.3) |
| Myocardial infarction | 351 (8.9%) | 45 (10.2%) | 1.2 (0.9-1.5) | 1.0 (0.7-1.4) |
| CABG/PCI | 242 (6.2%) | 24 (5.4%) | 0.9 (0.6-1.2) | 0.8 (0.5-1.3) |
| Atrial fibrillation/flutter | 417 (10.6%) | 41 (9.3%) | 0.9 (0.6-1.2) | 1.0 (0.7-1.5) |
| Coronary artery stenosis | 267 (7.5%) | 48 (12.9%) | 1.8 (1.4-2.3) | 1.7 (1.2-2.3) |
| Cardiac valvular disease | 184 (4.7%) | 23 (5.2%) | 1.1 (0.5-2.5) | 1.4 (0.6-3.4) |
| Heart failure | 93 (2.4%) | 26 (5.9%) | 2.6 (1.6-4.2) | 2.5 (1.5-4.4) |
| CAC total score | 0.0 (0.0-26.0) | 1.0 (0.0-75.0) | - | - |
| CAC category |  |  |  |  |
| 0 | 2,036 (51.8%) | 201 (45.4%) | 1 (Ref) | 1 (Ref) |
| 1-100 | 1,017 (25.9%) | 111 (25.1%) | 1.1 (0.8-1.4) | 1.1 (0.8-1.4) |
| >100 | 526 (13.4%) | 93 (21.0%) | 1.8 (1.4-2.3) | 1.6 (1.2-2.1) |
| Missing | 354 (9.0%) | 38 (8.6%) | - | - |

Data presented as frequency (%) and associations as odds ratio (95% confidence interval) analyzed using logistic regression.

^*^Adjusted for age, sex, smoking history, pack-years of smoking, highest completed education, and body mass index (when appropriate).

*Abbreviations:* please see Table S1.

**Table S29.** Population attributable fractions of breathlessness related to underlying medical conditions in people with self-reported cardiorespiratory disease

| **Condition** | **PAF (95% CI)** |
| --- | --- |
| Stress | 0.534 (-0.250-0.826) |
| Overweight and obesity | 0.463 (0.390-0.527) |
| Depression | 0.151 (0.113-0.187) |
| Respiratory disease | 0.144 (-0.076-0.319) |
| CAL | 0.142 (0.109-0.173) |
| Emphysema (on HRCT) | 0.082 (0.025-0.135) |
| Chronic bronchitis | 0.067 (0.030-0.103) |
| Restrictive spirometry pattern | 0.037 (0.022-0.051) |
| Other respiratory disease | 0.029 (-0.001-0.059) |
| Chronic rhinosinuitis | -0.021 (-0.073-0.028) |
| Asthma | -0.041 (-0.096-0.010) |
| CAC | 0.078 (-0.018-0.165) |
| Cardiac disease | 0.065 (-0.014-0.138) |
| Coronary artery stenosis | 0.043 (0.012-0.073) |
| Heart failure | 0.029 (0.010-0.049) |
| Cardiac valvular disease | 0.014 (-0.023-0.049) |
| Atrial fibrillation/flutter | 0.002 (-0.028-0.032) |
| Ischemic heart disease | -0.008 (-0.039-0.022) |
| Myocardial infarction | -0.004 (-0.033-0.024) |
| CABG/PCI | -0.009 (-0.030-0.012) |
| Anemia | 0.021 (0.000-0.041) |
| Peripheral arterial insufficiency | 0.004 (-0.002-0.010) |

For explanations and abbreviations, see Table 3 in the main article.

*Adjusted for age, sex, smoking history, pack-years, highest education and BMI (except when BMI is analyzed as factor).

**References**

1. Rosengren A, Hawken S, Ounpuu S, Sliwa K, Zubaid M, Almahmeed WA, Blackett KN, Sitthi-amorn C, Sato H, Yusuf S. Association of psychosocial risk factors with risk of acute myocardial infarction in 11119 cases and 13648 controls from 52 countries (the INTERHEART study): case-control study. *Lancet* 2004: 364(9438): 953-962.

2. Public Health Agency of Sweden. Hälsa på lika villkor. 2012. Available at: https://[www.folkhalsomyndigheten.se/](http://www.folkhalsomyndigheten.se/). Accessed 4 Dec 2018.

3. Rosengren A, Tibblin G, Wilhelmsen L. Self-perceived psychological stress and incidence of coronary artery disease in middle-aged men. *Am J Cardiol* 1991: 68(11): 1171-1175.

4. World Health Organization (WHO). Haemoglobin concentrations for the diagnosis of anaemia and assessment of severity. Vitamin and Mineral Nutrition Information System. WHO/NMH/NHD/MNM/11.1. Geneva: World Health Organization; 2011 (<http://www.who.int/vmnis/indicators/haemoglobin.pdf)>. [Assessed 3 Apr 2023].
